# Perinatal health outcomes of offspring of internal migrant women according to human development index: a registry-based cohort study of over 10 million live births from Brazil

**DOI:** 10.1016/j.lana.2025.101020

**Published:** 2025-02-12

**Authors:** Thiago Cerqueira-Silva, Enny S. Paixao, Ila R. Falcao, Joanna M.N. Guimarães, Laura C. Rodrigues, Alisson Baribieri, Ibrahim Ababukar, Mauricio L. Barreto, Julia M. Pescarini

**Affiliations:** a Faculty of Epidemiology and Population Health, London School of Hygiene & Tropical Medicine, London, United Kingdom; b Laboratório de Medicina e Saúde Pública de Precisão - Fundação Oswaldo Cruz - Salvador, Brazil; c Centro de Integração de Dados e Conhecimentos para Saúde (Cidacs), Fundação Oswaldo Cruz, Salvador, Brazil; d Centro de Desenvolvimento e Planejamento Regional (Cedeplar), Universidade Federal de Minas Gerais (UFMG), Belo Horizinte, Brazil; e Faculty of Population Health Sciences, University College London (UCL), London, United Kingdom

**Keywords:** Migrant health, Internal migration, Administrative data, Perinatal health, Neonatal mortality

## Abstract

**Background:**

Migration, driven by factors like poverty, violence, and natural disasters, is a key social determinant of health. While international migrants often have worse perinatal outcomes, research on perinatal health differences between internal migrants and non-migrants remains limited. We aimed to determine whether the offspring of women who migrate within Brazil experience poorer perinatal outcomes than those of non-migrants, according to the Human Development Index (HDI) of their municipalities of origin and destination.

**Methods:**

We used the CIDACS Birth Cohort, consisting of women applying for social programmes in the Unified Registry for Social Programmes *Cadastro Único* linked with live births and mortality registries. We included live births conceived from March 2010 to February 2018. Internal migrants were women who changed their state of residence from registration in CadUnico to the birth of the child. We derived risk ratios (RR) of migration's effect according to HDI of residence before and after migration using logistic regression.

**Findings:**

We included 10,184,021 births in the study, with 5.7% of these births from women who were internal migrants. The offspring of women who migrated to municipalities with equal/higher HDI (80% of migrations), exhibited a decreased risk of preterm births (RR: 0.94, 95% CI: 0.93–0.95), low birth weight (RR: 0.94, 95% CI: 0.92–0.95) and small for gestational age (RR: 0.92, 95% CI: 0.91–0.93), but higher risk of congenital abnormalities (RR: 1.14, 95% CI: 1.10–1.18). The offspring of women who migrated to municipalities with lower HDI had delayed access to healthcare and worse outcomes except for a lower risk of low birth weight (RR: 0.94, 95% CI: 0.92–0.96).

**Interpretation:**

Offspring of those migrating to municipalities with equal/higher HDI tend to have better perinatal outcomes, whereas migrants to lower HDIs have a similar pattern to non-migrant women.

**Funding:**

10.13039/501100000272NIHR, 10.13039/100010269Wellcome Trust, Royal Society.

**Research in context**

**Evidence before this study**

Most individuals migrate to seek improved living conditions; however, they often encounter significant challenges such as stigma, discrimination, and barriers to accessing healthcare. To assess the existing evidence on perinatal health outcomes among migrant populations, we conducted a comprehensive search of PubMed using the following terms: ((Transients and Migrants [MeSH Terms]) OR (Emigration and Immigration [MeSH Terms]) OR “displaced” OR “displacement”) AND (“Infant Mortality” [MeSH Terms] OR “Pregnancy Outcome” [MeSH Terms] OR “Prenatal Care” [MeSH Terms] OR “Maternal Health Services” [MeSH Terms] OR “Low Birth Weight” OR “Small for Gestational Age” OR “Premature Birth” OR “Apgar Score” OR “Congenital Abnormalities”). Our search yielded 1104 articles published since 1990. Of these, 11 studies (4 from China, 3 from Sub-saharian Africa, 2 from the United States (US), 1 from Guatemala and 1 from Myanmar conducted specifically examined perinatal health outcomes among internal migrant women in comparison to non-migrants. The findings from these studies are mixed. Seven studies reported that internal migration was associated with improved perinatal outcomes, including lower rates of preterm birth and low birth weight, as well as increased utilisation of health services. Conversely, two studies indicated poorer outcomes for migrant women, such as a higher risk of under-five mortality and lower healthcare utilisation. The remaining two studies found no significant differences between migrant and non-migrant women in terms of perinatal outcomes. Notably, none of the studies adjusted for differences in the level of social or economic development of origin and destination of migrants. Additionally, only one focused on Latin America, and involved fewer than 2000 women, highlighting a significant knowledge gap in this region.

**Added value of this study**

Our research provides compelling evidence on the effects of internal migration by analysing a large, representative Brazilian cohort of over 10 million women using two study designs: a cohort and a sibling study. Unlike previous research, our analysis accounted for differences in the Human Development Index (HDI) between migrants' origin and destination municipalities. We found that women who moved to municipalities with higher HDI had lower rates of preterm birth and small for gestational age newborns. Women who migrated to cities with lower HDI, on the other hand, had adverse perinatal outcomes at rates comparable to those of non-migrant women. Furthermore, our findings show that the positive effects of migration are concentrated in areas with higher HDI and more nurses per capita.

**Implications of all the available evidence**

This study adds to the existing body of literature on the perinatal health of migrant women by providing a more detailed and nuanced understanding of the impact of internal migration. In addition, our study is the first of its kind in the context of Latin America. Our findings suggest that key aspects in the destination municipality (e.g., economic and health infrastructure or quality of healthcare) are critical in determining perinatal outcomes for migrant women, reinforcing the idea that upscale migration can lead to better health whilst downscale migration can lead to poorer outcomes. These findings highlight the need for additional research into the motivations for migration and post-migration living conditions that influence perinatal health outcomes.

## Introduction

Migration is a social determinant of health, as people migrate to seek better living conditions, to escape poverty, violence, and natural disasters, among other factors.^1^ Evidence indicates that international migrants typically experience better health and lower overall mortality than those who remain in their home country and the local population at their destination. This phenomenon is known as the “healthy migrant effect”. However, migration itself can have long-lasting consequences on someone's physical and mental health,^1^ and migrants often encounter discrimination and challenges in accessing healthcare systems in their new locations.^2^ Among pregnant international migrants, these challenges can lead to worse perinatal health outcomes,^3^ such as increased risks of stillbirth, perinatal and neonatal mortality and delayed or poorer access to prenatal care than native-born.^4^, ^5^, ^6^ However, many of these effects are modified by factors such as the migrant's origin, educational level, time since arrival and study setting,^2^^,^^3^^,^^5^^,^^6^ and they reflect both the characteristics of migration and the socioeconomic conditions and access to healthcare available to migrants in the place of arrival.

In low-and middle-income countries (LMICs), rural-to-urban migrants often experience poorer perinatal outcomes and higher maternal or child mortality rates than non-migrants,^7^, ^8^, ^9^ which is likely to reflect both the characteristic of migration and the living and healthcare conditions in the place of arrival. While individual social networks can play a key role in one's decision to migrate,^10^ after migrating, individuals can lose social support and experience increased poverty and increased anxiety, resulting in adverse mental and perinatal mental health outcomes.^11^^,^^12^ However, the understanding of the differences in perinatal health outcomes between internal migrants and non-migrants is limited.

Brazil has a universal healthcare system, but there are significant disparities in service quality, particularly in poorer areas that struggle to meet local healthcare demands. As a result, some residents rely on healthcare infrastructure from other areas.^13^ In Brazil, according to the 2010 Census, there are approximately 28 million lifetime internal migrants (i.e., defined as individuals living in a different state from the one in which they were born).^14^ Although the reasons for migration can be diverse, in Brazil, recent migration fluxes continue to be towards wealthy areas of Brazil, with new migrations fluxes to growing municipalities in the North, central-west and Northeast, intensifying short-distance flows (i.e., within states).^15^ However, migration fluxes in Brazil are likely to be largely underestimated due to migrations of return—when individuals return to their original place of birth. Despite this large migrant population,^16^ there is limited understanding in Brazil and Latin America regarding the impact of the migration process on internal populations, according to an individual's origin and destination, and on whether it affects perinatal care compared to before migration or non-migrant populations.

Here, by using linked data of the poorest half of Brazilians applying for social programmes in the country,^17^ we (i) evaluated the characteristics of women giving birth who are internal migrants in Brazil, (ii) investigated whether offspring of internal migrant women in Brazil have poorer perinatal outcomes compared to non-migrant Brazilians and (iii) investigated whether the risk of poor perinatal outcomes in offspring of internal migrant women differs based on their migration to municipalities with equal/higher or lower Human Development Index (HDI) than their original municipalities.

## Methods

### Study design and datasets

We conducted a cohort study using live births in Brazil from the CIDACS Birth Cohort from 1st January 2011 to 31st December 2018.^18^ This cohort was built from harmonised and compiled data on individuals first applying for social programmes in the Unified Registry for Social Programmes *Cadastro Único–*CadUnico) to create an open cohort of now over 130 million individuals named the 100 Million Brazilian Cohort. The 100 Million Brazilian Cohort^17^ was then linked with nationwide live births from the Live Births Information System (*Sistema de Informação de Nascidos Vivos*—SINASC) and mortality registries from the Mortality Information System (*Sistema de Informação em Mortalidade*—SIM) to create the birth cohort. The linkage was conducted by an expert team of researchers and the unidentified database provided in a haven for analysis. The specific information regarding the linkage of three databases was previously published.^19^

For this analysis, we extracted detailed individual self-reported socioeconomic and demographic information of women at the time of enrolment/application in CadUnico, which for this study varied from 1st January 2011 to 31st December 2018. From SINASC, we extracted demographic information on the mother at the time of delivery and information on prenatal care and on the live birth. From SIM we extracted information on the day and cause of death among live births.

### Participants

We excluded (i) births that took place prior to 22 weeks or after 44 weeks of gestation, or with a birthweight below 500*g*; (ii) twins or multiple births; (iii) births without a documented place of residence; (iv) births to women aged less than 10 years old and to women aged more than 49 years; and (v) births to women born outside Brazil; (vi) births with data inconsistencies, such as conception dates of consecutive births occurring less than 220 days apart or records with different birth dates of the mother across databases. To mitigate fixed cohort bias,^20^ we looked specifically at live births from pregnancies with estimated conception dates between March 1, 2010 and February 1, 2018. Given a gestation period of 40 weeks, pregnancies that commenced on February 1, 2018 would result in deliveries on November 8, 2018. This timeline provided a minimum of 28 days for post-birth follow-up. The estimated date of conception was calculated as the birth date minus the gestational age, which was determined using either ultrasound, last menstrual period or clinical examination.

### Exposure and covariates

The main exposure was whether the mother was an internal migrant or not. The definition of an internal migrant was a woman who moved from the State where she lived when enrolled in CadUnico. Although migration was defined at the State level, we used the municipality of registration and the municipality of birth Human Development Index (HDI) to stratify the exposure.^21^ The HDI is calculated by combining three key dimensions: health, measured by life expectancy at birth; education, assessed through average years of schooling for adults and expected years of schooling for children; and standard of living, represented by gross national income per capita. Each dimension is normalised on a scale from 0 to 1, and then averaged to produce the HDI. We stratified the migration group based on the difference in HDI between the municipality of enrolment in the CadUnico and the municipality of delivery of the newborn, each pregnancy was classified as non-migrant, migration to an equal/higher HDI or migration to lower HDI. We further stratified each category (lower or equal/higher HDI) by the difference in HDI ≤0.1 and > 0.1. Supplementary Figure S1 shows the geographic and administrative divisions of Brazil and the distribution of HDI across the country. Supplementary Figure S2 shows the distribution of differences in HDI across the migrant groups.

The variables to include in the outcome model were selected based on the disjunctive cause criterion, that is any pre-exposure covariate that is a cause of the exposure, or the outcome, or both.^22^ Our assumptions regarding the relationships among the variables are shown in Supplementary Figure S3.

The propensity score model included: date of registration in CadUnico, state of residency, state of cohort registry different from birth, location of household (rural or urban area), material of household (masonry/brick, coated or uncoated Taipa, wood, other), water system (public system, well or other), waste disposal/garbage collection, HDI of the municipality, age, education level, race/ethnicity. All variables were measured before the pregnancy.

The outcome model included: the age of the mother, state of residence, number of antenatal visits, education level, year of conception, number of previous pregnancies (0 or ≥1), receipt of conditional cash transfer benefit, marital status, and previous foetal loss. All variables were measured at the time of the child's birth.

### Outcomes

We examined perinatal outcomes that could be associated with access and quality of healthcare, including (i) timely initiation of antenatal care (at least one antenatal appointment within the first trimester), (ii) preterm (gestational age <37 weeks), (iii) low birth weight (LBW; birth weight <2500*g*), (v) small for gestational age (SGA; weight below the 10th percentile for gestational age and sex), (vi) low Apgar score (Apgar score below 7 at 5 min after birth), (vii) congenital anomaly at birth, and (viii) neonatal mortality (death up to 28 days of life). We categorised the size of newborns as small, appropriate, or large for their gestational age by utilising sex-specific curves from the INTERGROWTH-21st Consortium for singleton births.^23^

### Statistical analysis

We assessed the effect of migration on the offspring of mothers according to the HDI of municipalities they migrated from and to by comparing each group to the offspring of women who did not migrate. We utilised inverse probability weights (IPW) derived from a generalised boosted model to calculate the probability of being a migrant.^24^ Separate weights were calculated for each subgroup comparison (i.e., migration to municipalities with equal/higher HDI or lower HDI vs non-migrants) using the following baseline socioeconomic covariates recorded at baseline. The main objective was to examine the marginal average treatment effect on the treated (ATT) of the outcomes. The treatment effect sizes, and their corresponding 95% confidence intervals (95% CI) were calculated using a weighted logistic regression model. We included cluster robust (sandwich) standard errors (SEs) to account for multiple births from the same mother. We calculated the risk ratio (RR) by adjusting for weights and incorporating variables from the live birth database (SINASC). The RR was derived using regression standardisation and SE was derived using the delta method.^25^ Missing data in the covariates was addressed in the main analysis by utilising a missing indicator.

### Robustness checks: migration between pregnancies using a sibling analysis

To assess the robustness of our results and control for potential unmeasured confounding related to the mothers, we implemented a sibling design, restricting the sample to women with more than one live birth during the study period. We used the same set of inclusion and exclusion criteria of the primary analysis. Each woman's live birth was classified according to mother condition: non-migrant, migration to an equal/higher HDI or migration to lower HDI, the reference group was the non-migrant. We employed conditional logistic regression to calculate the conditional Odds Ratio (OR) and incorporated the mother's age, year of conception, order of birth, education level, and marital status as covariates in the outcome model.

### Sensitivity analysis

We also conducted three sensitivity analyses. First, we estimated the RR for the association between migration and perinatal outcomes using a continuous difference in HDI (i.e., 0.1 intervals) to see if the magnitude of the effect varies when differences in HDI of the municipality of origin and arrival differ more. Second, to verify the potential introduction of bias due to classifying missing data as an indicator, we assumed that missing could be at random and used multiple imputation by chained equations using a fully conditional specification with five imputed datasets using the within approach to combine the propensity scores. Third, to understand how much the effects could be related to the availability of healthcare in the municipality at birth, we further (i) calculated the effects of migration accounting for the going to municipalities with equal/higher or lower standardised number of nurses per 1000 inhabitants for the year of birth^26^; and (ii) included the HDI of the municipality of residence at birth as a covariate in the outcome model, modelling as a spline. The analysis was conducted in R 4.3.1, using the packages “*WeightIt”, “fixest”* and “*marginaleffects*”.

### Ethics

The CIDACS Birth Cohort and this study received approval from the ethics committees at Instituto Gonçalo Muniz—Oswaldo Cruz Foundation (Ref 3.551.787/2021 and 4.534.397/2021); and from The London School of Hygiene and Tropical Medicine (Ref 22,817/2021 and 22,771/2021).

### Role of funding source

The funders of the study had no role in study design, data collection, data analysis, data interpretation, or writing of the report.

## Results

**Fig. 1:**
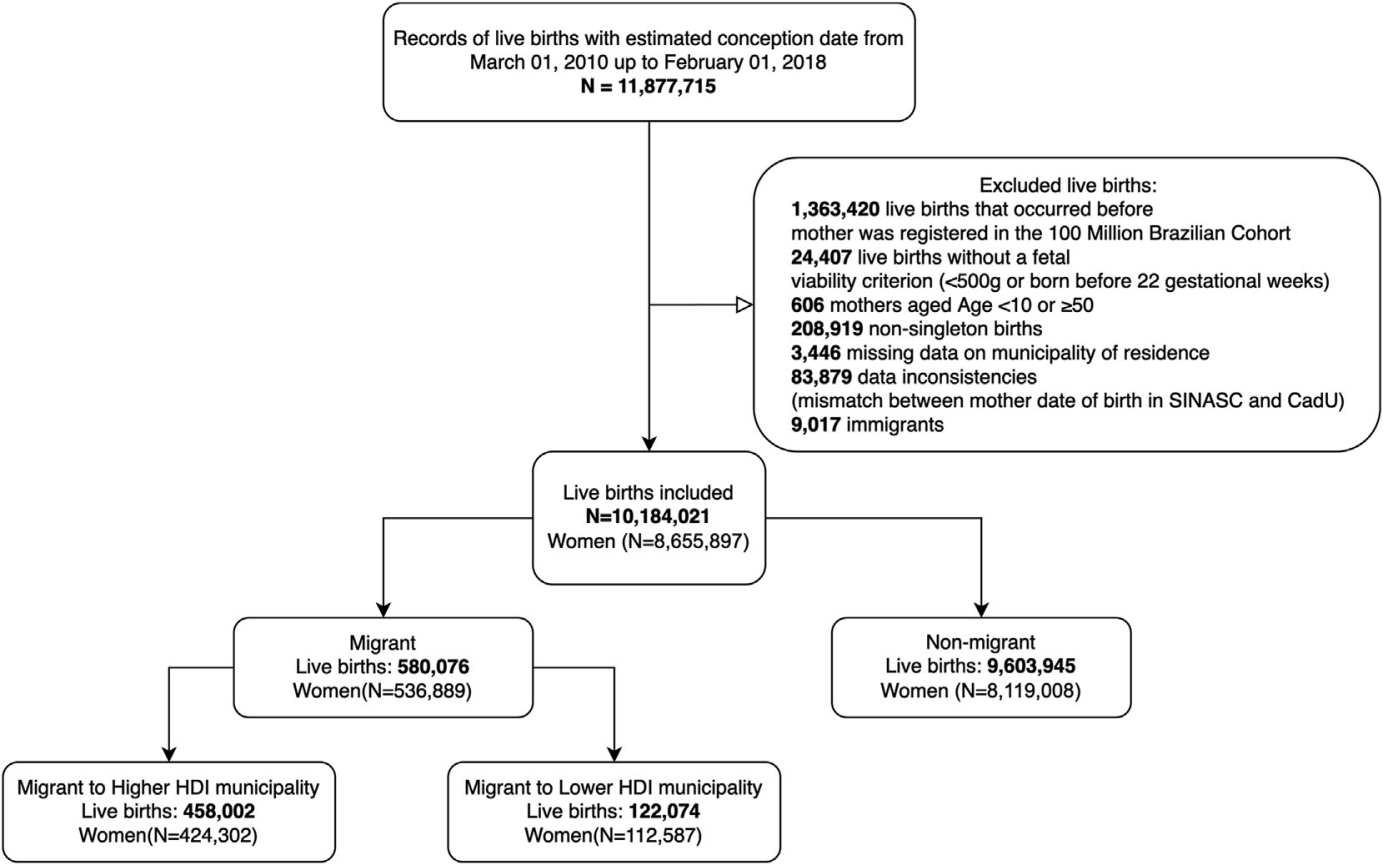
**Selection of the study participants**.

**Fig. 2:**
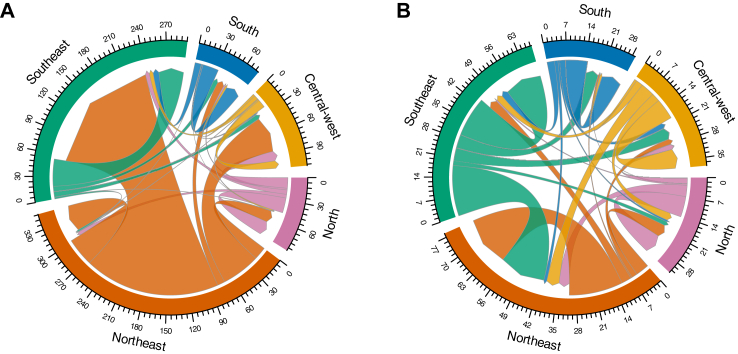
**Fluxes of migrant women between cohort entry and childbirth among those who moved to a equal/higher HDI municipality (A) or to a lower HDI municipality (B) by region of the country**. The numbers are expressed in N∗1000.

The study included 10,184,021 live births from 8,655,897 women. Among live births, 9,603,945 (94.3%) were from non-migrant women, while 580,076 (5.7%) were from internal migrant women. Most internal migrant women (458,002; 78.9%) moved to municipalities with an equal/higher HDI (Fig. 1). At baseline, most women who migrated to municipalities with higher HDI resided in the Northeast region (66.0%) and in rural areas (39.3%) compared to non-migrants (24.6%) and migrants to lower HDI municipalities (15.2%) (Table 1, Fig. 2). Conversely, women moving to municipalities with lower HDI were mainly from urban areas (84.8%) and lived in the Southeast region (36.0%). Regarding sanitation conditions, migrants to higher HDI areas had lower access to basic sanitation services, such as sewage systems and waste collection at baseline, compared to those moving to lower HDI municipalities and non-migrants (Table 1). The inverse probability weighting resulted in a satisfactory balance across all baseline variables (standardised mean differences <0.1) (Supplementary Figure S4).

**Table 1:** Sociodemographic and economic characteristics of live births of internal migrant women moving to higher/equal HDI or lower HDI and non-migrant women.

Characteristic	Equal/Higher^a^ HDI	Lower HDI	Non-migrants	Overall
	N = 458,002	N = 122,074	N = 9,603,945	N = 10,184,021
**No. women**	424,302	112,587	8,119,008	8,655,897
**Mother's demographic characteristics at cohort baseline**				
Age years; median (IQR)	16 (12, 20)	17 (12, 23)	17 (12, 22)	17 (12, 22)
Education level				
No school	18,509 (4.0%)	5889 (4.8%)	494,408 (5.1%)	518,806 (5.1%)
Nursery	3525 (0.8%)	1318 (1.1%)	108,560 (1.1%)	113,403 (1.1%)
Infant school (learning to read/write)	4061 (0.9%)	985 (0.8%)	105,725 (1.1%)	110,771 (1.1%)
Middle school 1	121,616 (27%)	33,895 (28%)	2,626,528 (27%)	2,782,039 (27%)
Middle school 2	150,967 (33%)	38,830 (32%)	2,972,815 (31%)	3,162,612 (31%)
High school	58,300 (13%)	16,307 (13%)	1,676,979 (17%)	1,751,586 (17%)
Higher education	1624 (0.4%)	836 (0.7%)	80,214 (0.8%)	82,674 (0.8%)
Missing	99,400 (22%)	24,014 (20%)	1,538,716 (16%)	1,662,130 (16%)
Race/ethnicity				
White	97,272 (21%)	37,461 (31%)	2,707,899 (28%)	2,842,632 (28%)
Black	26,163 (5.7%)	8001 (6.6%)	716,756 (7.5%)	750,920 (7.4%)
Mixed	283,107 (62%)	65,527 (54%)	5,381,008 (56%)	5,729,642 (56%)
Asian	1876 (0.4%)	413 (0.3%)	34,890 (0.4%)	37,179 (0.4%)
Indigenous	1591 (0.3%)	629 (0.5%)	90,179 (0.9%)	92,399 (0.9%)
Missing	47,993 (10%)	10,043 (8.2%)	673,213 (7.0%)	731,249 (7.2%)
**Mother's living conditions at cohort baseline**				
Water system				
Public system	246,161 (54%)	86,513 (71%)	6,334,017 (66%)	6,666,691 (65%)
Water well	128,157 (28%)	19,038 (16%)	2,032,063 (21%)	2,179,258 (21%)
Other	47,667 (10%)	7315 (6.0%)	782,768 (8.2%)	837,750 (8.2%)
Missing	36,017 (7.9%)	9208 (7.5%)	455,097 (4.7%)	500,322 (4.9%)
Material of the household				
Masonry/brick	263,638 (58%)	85,488 (70%)	6,579,064 (69%)	6,928,190 (68%)
Coated Taipa	23,914 (5.2%)	2460 (2.0%)	295,700 (3.1%)	322,074 (3.2%)
Uncoated Taipa	22,677 (5.0%)	2187 (1.8%)	291,284 (3.0%)	316,148 (3.1%)
Wood	48,415 (11%)	16,980 (14%)	1,325,113 (14%)	1,390,508 (14%)
Other	63,329 (14%)	5752 (4.7%)	657,629 (6.8%)	726,710 (7.1%)
Missing	36,029 (7.9%)	9207 (7.5%)	455,155 (4.7%)	500,391 (4.9%)
Location of household				
Urban	258,287 (56%)	96,584 (79%)	6,988,461 (73%)	7,343,332 (72%)
Rural	167,355 (37%)	17,286 (14%)	2,275,200 (24%)	2,459,841 (24%)
Missing	32,360 (7.1%)	8204 (6.7%)	340,284 (3.5%)	380,848 (3.7%)
Household type				
Permanent (independent of ownership)	406,915 (89%)	104,930 (86%)	8,661,260 (90%)	9,173,105 (90%)
Improvised (e.g., shacks)	2063 (0.5%)	603 (0.5%)	79,747 (0.8%)	82,413 (0.8%)
Collective (household occupied by multiple families)	235 (<0.1%)	104 (<0.1%)	9359 (<0.1%)	9698 (<0.1%)
Other	8965 (2.0%)	5022 (4.1%)	267,556 (2.8%)	281,543 (2.8%)
Missing	39,824 (8.7%)	11,415 (9.4%)	586,023 (6.1%)	637,262 (6.3%)
Waste disposal/garbage collection				
Collected direct/indirectly	237,331 (52%)	93,300 (76%)	6,689,097 (70%)	7,019,728 (69%)
Burned or buried	116,056 (25%)	13,381 (11%)	1,700,541 (18%)	1,829,978 (18%)
Open air dump	63,754 (14%)	4589 (3.8%)	645,713 (6.7%)	714,056 (7.0%)
Other	4853 (1.1%)	1600 (1.3%)	113,453 (1.2%)	119,906 (1.2%)
Missing	36,008 (7.9%)	9204 (7.5%)	455,141 (4.7%)	500,353 (4.9%)
State of cohort registry different from birth	86,191 (19%)	51,579 (42%)	942,759 (9.8%)	1,080,529 (11%)
Missing	10,592 (2.3%)	4211 (3.4%)	717,876 (7.5%)	732,679 (7.2%)
Municipality HDI—CadUnico enter	0.61 (0.57, 0.67)	0.75 (0.70, 0.79)	0.71 (0.62, 0.76)	0.70 (0.61, 0.76)
CadUnico—Register geographical region				
North	47,549 (10%)	14,201 (12%)	1,192,881 (12%)	1,254,631 (12%)
Northeast	302,195 (66%)	30,409 (25%)	3,666,218 (38%)	3,998,822 (39%)
Southeast	53,587 (12%)	43,899 (36%)	2,980,673 (31%)	3,078,159 (30%)
South	32,605 (7.1%)	14,626 (12%)	1,094,252 (11%)	1,141,483 (11%)
Central west	22,066 (4.8%)	18,939 (16%)	669,921 (7.0%)	710,926 (7.0%)
**Mother's characteristics at pregnancy and birth**				
Age at pregnancy–years; median (IQR)	24 (20, 28)	24 (20, 30)	24 (20, 29)	24 (20, 29)
Age at pregnancy				
10–17	38,077 (8.3%)	14,213 (12%)	1,116,956 (12%)	1,169,246 (11%)
18–24	207,806 (45%)	49,363 (40%)	3,971,162 (41%)	4,228,331 (42%)
25–29	126,238 (28%)	27,593 (23%)	2,222,512 (23%)	2,376,343 (23%)
30–34	59,698 (13%)	18,862 (15%)	1,418,534 (15%)	1,497,094 (15%)
35–49	26,183 (5.7%)	12,043 (9.9%)	874,781 (9.1%)	913,007 (9.0%)
Number of prenatal appointments				
None	6797 (1.5%)	2254 (1.8%)	178,473 (1.9%)	187,524 (1.8%)
1–3	30,455 (6.6%)	10,947 (9.0%)	769,666 (8.0%)	811,068 (8.0%)
4–6	118,771 (26%)	38,856 (32%)	2,812,583 (29%)	2,970,210 (29%)
≥7	299,059 (65%)	69,318 (57%)	5,789,567 (60%)	6,157,944 (60%)
Missing	2920 (0.6%)	699 (0.6%)	53,656 (0.6%)	57,275 (0.6%)
Pregnancy—Geographical region				
North	39,944 (8.7%)	17,567 (14%)	1,192,881 (12%)	1,250,392 (12%)
Northeast	46,442 (10%)	47,865 (39%)	3,666,218 (38%)	3,760,525 (37%)
Southeast	240,823 (53%)	23,688 (19%)	2,980,673 (31%)	3,245,184 (32%)
South	43,264 (9.4%)	14,313 (12%)	1,094,252 (11%)	1,151,829 (11%)
Central west	87,529 (19%)	18,641 (15%)	669,921 (7.0%)	776,091 (7.6%)
Education level at birth				
None	1983 (0.4%)	1214 (1.0%)	74,186 (0.8%)	77,383 (0.8%)
1–3	15,409 (3.4%)	6297 (5.2%)	416,432 (4.3%)	438,138 (4.3%)
4–7	112,798 (25%)	35,341 (29%)	2,467,218 (26%)	2,615,357 (26%)
8–11	297,757 (65%)	69,931 (57%)	5,920,598 (62%)	6,288,286 (62%)
≥12	25,103 (5.5%)	7134 (5.8%)	567,691 (5.9%)	599,928 (5.9%)
Missing	4952 (1.1%)	2157 (1.8%)	157,820 (1.6%)	164,929 (1.6%)
Pregnancy year				
2010	18,484 (4.0%)	6918 (5.7%)	513,054 (5.3%)	538,456 (5.3%)
2011	56,015 (12%)	14,850 (12%)	1,103,165 (11%)	1,174,030 (12%)
2012	82,786 (18%)	21,130 (17%)	1,358,224 (14%)	1,462,140 (14%)
2013	107,810 (24%)	28,230 (23%)	1,518,094 (16%)	1,654,134 (16%)
2014	101,261 (22%)	26,029 (21%)	1,584,016 (16%)	1,711,306 (17%)
2015	41,694 (9.1%)	10,882 (8.9%)	1,197,776 (12%)	1,250,352 (12%)
2016	22,095 (4.8%)	6116 (5.0%)	1,085,749 (11%)	1,113,960 (11%)
2017	25,603 (5.6%)	7231 (5.9%)	1,144,482 (12%)	1,177,316 (12%)
2018	2254 (0.5%)	688 (0.6%)	99,385 (1.0%)	102,327 (1.0%)
Number of previous pregnancies				
0	177,104 (39%)	35,219 (29%)	3,055,809 (32%)	3,268,132 (32%)
≥1	264,562 (58%)	81,642 (67%)	6,094,994 (63%)	6,441,198 (63%)
Missing	16,336 (3.6%)	5213 (4.3%)	453,142 (4.7%)	474,691 (4.7%)
Civil status				
Single	227,203 (50%)	52,325 (43%)	4,555,710 (47%)	4,835,238 (47%)
Common law—Marriage	140,493 (31%)	44,108 (36%)	2,728,078 (28%)	2,912,679 (29%)
Married	82,428 (18%)	22,779 (19%)	2,107,086 (22%)	2,212,293 (22%)
Divorced	3343 (0.7%)	976 (0.8%)	81,305 (0.8%)	85,624 (0.8%)
Widowed	562 (0.1%)	246 (0.2%)	16,696 (0.2%)	17,504 (0.2%)
Missing	3973 (0.9%)	1640 (1.3%)	115,070 (1.2%)	120,683 (1.2%)
Previous foetal loss	77,591 (17%)	23,743 (19%)	1,659,741 (17%)	1,761,075 (17%)
Missing	29,302 (6.4%)	10,871 (8.9%)	869,872 (9.1%)	910,045 (8.9%)
Municipality HDI – Pregnancy	0.77 (0.73, 0.81)	0.67 (0.60, 0.72)	0.71 (0.63, 0.76)	0.71 (0.63, 0.76)
HDI difference between municipalities				
≤0.1	160,317 (35%)	86,243 (71%)	–	–
>0.1	297,685 (65%)	35,831 (29%)	–	–
Ever received conditional cash transfer (yes)	429,048 (94%)	112,567 (92%)	8,692,478 (91%)	9,234,093 (91%)
**Characteristics of the newborn**				
Low birth weight (<2500g)	30,932 (6.8%)	683,735 (7.1%)	8167 (6.7%)	722,834 (7.1%)
Preterm birth (<37 weeks)	44,185 (9.6%)	13,273 (11%)	1,036,944 (11%)	1,094,402 (11%)
Low Apgar 5’ (<7)	4832 (1.1%)	1338 (1.1%)	103,853 (1.1%)	110,023 (1.1%)
Missing	5208 (1.1%)	4356 (3.6%)	309,291 (3.2%)	318,855 (3.1%)
Size for gestational age				
Adequate	349,835 (76%)	90,402 (74%)	7,185,031 (75%)	7,625,268 (75%)
Small	35,816 (7.8%)	10,185 (8.3%)	798,097 (8.3%)	844,098 (8.3%)
Large	72,351 (16%)	21,487 (18%)	1,620,817 (17%)	1,714,655 (17%)
Delayed antenatal care (Start >3rd month)	99,062 (22%)	30,904 (25%)	2,292,782 (24%)	2,422,748 (24%)
Missing	27,559 (6.0%)	7729 (6.3%)	596,329 (6.2%)	631,617 (6.2%)
Congenital abnormalities	4414 (1.0%)	867 (0.7%)	74,253 (0.8%)	79,534 (0.8%)
Missing	12,600 (2.8%)	3919 (3.2%)	240,028 (2.5%)	256,547 (2.5%)
Neonatal death (≤28 days)	2768 (0.6%)	64,062 (0.7%)	864 (0.7%)	67,694 (0.7%)

HDI, Human Development Index; AGA, Adequate for gestational age; SGA, Small for gestational age; LGA, Large for gestational age.

The percentages may not sum to 100% due to rounding values.

a The group equal/higher contains 1439 (0.24%) cases of no difference (equal HDI).

**Fig. 3:**
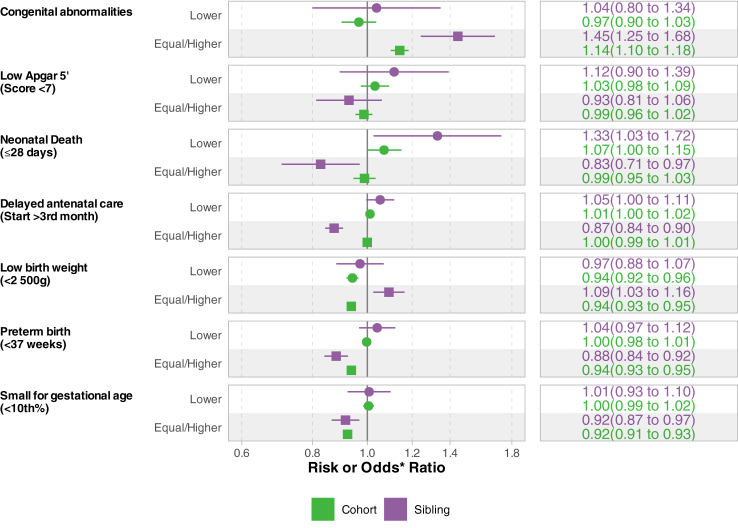
**Risk Ratio of perinatal outcomes of women according to migration to equal/higher or lower Human Development Index municipalities by study design**. The cohort study is adjusted for date of registration in CadUnico, state of residency, state of cohort registry different from birth, location of household (rural or urban area), material of household, water system, waste disposal/garbage collection, HDI of the municipality origin, age, education level, race/ethnicity (Propensity score–inverse probability weighting adjustment, variables measured at CadUnico registration) and age of the mother, state of residence, number of antenatal visits, education level, year of conception, parity, receipt of conditional cash transfer benefit, marital status, and previous foetal loss (Outcome model, variables measured at each childbirth). The sibling study is adjusted for the age of the mother, year of conception, education level, order of birth and marital status. (measured at each childbirth). Reference group in both study designs is non-migrant women. ∗Values for the sibling study are presented as Odds ratio.

Compared with non-migrant women, live births to women migrating to equal/higher HDI municipalities had a lower risk of being LBW (RR: 0.94 [95% CI 0.93–0.95]), preterm (RR: 0.94 [95% CI 0.93–0.95]), and SGA (RR: 0.92 [95% CI 0.91–93]) (Fig. 3-cohort study). However, they presented a higher risk for congenital abnormalities (RR: 1.14 [95% CI 1.10–1.18]). The most common causes of congenital abnormalities were congenital malformations and deformations of the musculoskeletal system in all groups, however, among migrants to higher HDI municipalities, the prevalence of congenital malformations of the circulatory system was higher than that in migrants to lower HDI and non-migrant (0.13%, 0.04% and 0.06% respectively) (Supplementary Table S1). When looking at continuous differences in HDI, we observed a suggestive trend in the effect especially for congenital abnormalities, preterm birth and SGA (Supplementary Figure S4).

Births from women who migrated to lower HDI municipalities had similar risks of perinatal outcomes as non-migrant women, except for a lower risk of LBW (RR: 0.94 [95% CI 0.92–0.96]) and a higher risk of neonatal death (RR: 1.07 [95% CI 1.00–1.15) (Fig. 3). The analysis stratified by changes in HDI scores of municipalities (≤0.1 or >0.1) suggested dose-dependent behaviour, with the group migrating to municipalities with >0.1 higher HDI having lower risks of LBW (RR: 0.95 [95% CI 0.93–0.97]), prematurity (0.94 [0.92–0.95]), and small for gestational age (0.90 [0.89–0.92]) (Supplementary Table S2 and Supplementary Figure S5). In contrast, women who migrated to municipalities with ≤0.1 lower HDI had a higher risk of SGA (RR: 1.05 [95% CI 1.02–1.09]) and a low Apgar 5’ score (1.12 [1.01–1.23]).

The sibling analysis supported the main results but showed larger effects overall and a substantially lower risk of delayed antenatal care (OR: 0.87 (95% CI: 0.84–0.90)) and neonatal death (OR: 0.83 [95% CI: 0.71–0.97]) for women migrating to equal/higher HDI municipalities compared to the live births of non-migrant women; while those migrating to municipalities with lower HDI had a higher risk of neonatal death (OR: 1.33 (95% CI: 1.03–1.72)). In addition, newborns from women who migrated to equal/higher HDI municipalities had a higher risk of being LBW (OR: 1.09 [95% CI 1.03–1.16]) (Fig. 3 and Supplementary Tables S3 and S4).

Our additional sensitivity analysis, utilising multiple imputation to account for missing data, yielded consistent results for all evaluated outcomes (Fig. 3 and Supplementary Table S5). Adjusting for HDI of the municipality of residence at birth showed a significant discordant result only for a low Apgar 5’ score. In the model adjusting for HDI, newborns of women who migrated to higher HDI areas had a higher risk (RR: 1.05 [95% CI 1.02–1.09]), while the model without adjusting for HDI did not show a significant result (Supplementary Table S6). Finally, the model classifying women by the difference in the HDI and number of nurses per 1000 inhabitants exhibited similar patterns to the main analysis, except that the increased risk of neonatal death for those migrating to lower HDI municipalities was present only when they also migrated to municipalities with a lower number of nurses (RR: 1.10 (95% CI: 1.01–1.21) (Supplementary Table S7).

## Discussion

This study investigated whether perinatal outcomes, including antenatal care, birth-related factors (i.e., congenital abnormalities, LBW, Apgar score, preterm birth, SGA) and neonatal mortality, are associated with internal migration in Brazil. We found that most internal migration in Brazil occurs towards more developed areas. Women who migrate to municipalities with higher HDI are more likely to have poorer socioeconomic conditions prior to migration than those moving to municipalities with lower HDI and non-migrants. We found that migration to municipalities with higher HDI is associated with advantages in perinatal health, whereas migration to municipalities with lower HDI presents certain disadvantages. Women migrating to more developed municipalities (i.e., those with higher HDI) presented a lower risk of LBW, preterm birth, and SGA, but a higher risk of congenital abnormalities in a dose-dependent pattern in terms of HDI scores. In contrast, women who migrated to less developed municipalities (i.e. those with lower HDI) had a lower risk of LBW only. Furthermore, in the sibling analysis, we found that migration to higher HDI was associated with a lower risk of delayed antenatal care and neonatal mortality. However, migration to municipalities with lower HDI was associated with a higher risk of delayed antenatal care and neonatal mortality.

Few studies have investigated the effects of internal migration on perinatal health outcomes worldwide.^7^^,^^8^^,^^27^, ^28^, ^29^, ^30^, ^31^, ^32^ Previous studies reported lower,^7^^,^^27^ higher^27^^,^^30^ or no differences^28^ in the use of maternal healthcare services among internal migrants compared to non-migrants, lower risk of preterm and low-birth-weight babies,^29^^,^^31^ and lower risk of child mortality.^8^ Importantly, whereas Guatemala rural-to-urban migrants experience greater prenatal care utilisation,^32^ in sub-Saharan Africa rural-to-rural but not all types of migration are associated with negative adaptation and poorer maternal healthcare utilisation, which is suggestively linked to lower availability and poorer healthcare services in rural compared to urban areas.^28^ In addition, a study evaluating internal migrants to Shanghai, China, reported a higher proportion of high-risk pregnancies (i.e. with comorbidities, complications, obstetric risk, among others) among internal migrants than among local mothers.^9^ This finding highlights the difficulties faced by internal migrants in accessing healthcare because the household registration system restricts access to healthcare and other public services outside their catchment area.

Our study differs from prior research by considering the variations in HDI between migrants' origin and destination municipalities. Our findings suggest that migrations relocating to a lower HDI municipality face major barriers due to the inherently poor quality of healthcare services, experiencing higher delays in accessing prenatal care than local mothers. Conversely, migrants to equal/higher HDI municipalities experience increased detection of congenital abnormalities. This finding is likely attributable to the availability of more structured healthcare and genetic services in wealthier municipalities and state capitals.^33^ The increased timely prenatal care and reduced risk of neonatal mortality, especially when mothers migrate to more developed municipalities with better healthcare infrastructure (i.e., more nurses per capita), reinforce the importance and consistency of the sibling design and indicate that migration could also be an adaptation strategy to improve health. However, we should be careful when interpreting these results as there is a possibility of ecological fallacy–i.e., the nurses' availability in the municipality of residence does not necessarily reflect the access and quality of the healthcare service provided to the individuals.

Prenatal care is essential for early detection and treatment of various conditions, including foetal growth restriction, chronic diseases (e.g., hypertension and gestational diabetes), infectious diseases (e.g., syphilis), and other gestational risk factors. Monitoring and identifying potential risks during the prenatal period are important for planning the timing of delivery, such as in a tertiary care centre, thereby increasing the chances of infant survival.^34^ The Brazilian Universal Healthcare System (*Sistema Único de Saúde*) provides free healthcare to all people and has significantly increased access to perinatal care in Brazil in the past 30 years.^35^ However, the decentralisation of funding and implementation of programmes aimed at improving perinatal care are dependent on contributions from Federal, State and municipal levels, which results in substantial variability in the quality of assistance across Brazil.^35^ Therefore, we hypothesise that the structure and organisation of local healthcare services can lead migrant mothers to municipalities with lower HDI to have a greater chance of delays in accessing care than migrant mothers to municipalities with higher HDI.

Delays in accessing prenatal care can have lifelong impacts on pregnancy, birth outcomes, and the early years of the child's life. In addition to access to healthcare, social networks at the place of arrival are important, so migrants can have access to housing, food and employment,^10^, ^11^, ^12^ and its absence can be particularly critical for pregnant women who require knowledge of the health system and security to seek and access timely prenatal care. The hypothesis that the improved or decreased access to healthcare could influence internal migrant health is also supported by the overall similar risk of neonatal death compared to non-migrants, but lower risk of neonatal mortality among internal migrants to lower HDI municipalities in the sibling design. These findings suggest potential improvements in mothers' access to healthcare after the migration process. We didn't find other studies comparing neonatal mortality according to different types of migration in other LMICs, but a study looking at mortality trajectories of children from birth to 14 years old in Kenya and Nigeria found migrant children to have overall better survival chances than non-migrants.^36^

The sibling analysis conducted in this study provides the best causal estimate of the true effect of migration. Once this study design can adjust to unmeasured confounders such as health-seeking behaviour, medical comorbidities, genetic factors etc., the effect will reflect the structural changes that the mother will have in their socioeconomic conditions and access to health care after migration.^37^^,^^38^ Despite the sibling study focusing only on a reduced proportion of our cohort (29%), the results are fairly consistent with the main analysis using the entire cohort, reinforcing our findings.

Our cohort also has some limitations. First, although nearly 14% of the Brazilian population are internal migrants, only 5% of the live births in our cohort were from internal migrant women. Second, the CIDACS Birth Cohort uses administrative data from people applying for social programmes and has limited generalizability to the poorest part of the Brazilian population. In addition, our administrative datasets have limited data on confounding factors for the association between migration and perinatal outcomes, such as the exact date and reason for the migration, and data on medical comorbidities or quality of the perinatal care provided. In addition, possible changes in socioeconomic status from enrolment in the cohort and the moment of birth that were driven by migration are important for further investigation but were not considered here. Although we differentiated migration based on differences in HDI from the place of origin to the destination, it is important to note that in the same way, migration can lead to changes in socioeconomic and health status, the initial health status at the origin can influence the odds of migrating and the health outcomes at the destination. Third, we lack data on stillbirths, and therefore we are unable to estimate whether migration can lead to losses of complicated pregnancies, increasing the number of births of “healthier” children when moving to municipalities with higher HDI. Similarly, terminating pregnancies with severe congenital problems that may pose a risk to the mother could be facilitated in municipalities with higher HDI.

In conclusion, our study extends the literature on how internal migration may impact perinatal health inequalities, especially in the contexts of Latin America and the Global South. Specifically, we found that migration can either be associated with poorer or improved perinatal health of migrant populations and that this is likely to depend on the characteristics of the place of origin and arrival, such as socioeconomic and health characteristics. Further research is needed to understand the drivers of migration and how migration can act as an adaptation strategy. Our study suggests that the place of arrival and, consequently, its socioeconomic characteristics and health systems are likely to play a key role in migrants' health.

## Contributors

TC-S and JMP conceptualised the study and wrote the manuscript's first draft. MLB secured data access. JMP supervised the data analysis. TC-S defined the methodology and conducted the formal analysis. All authors contributed to the writing, reviewing, and editing of the manuscript. JMP decided to submit the manuscript for publication. TC-S, ESP, and JMP accessed and verified the raw data in the study.

## Data sharing statement

The relevant data are available in the manuscript and the appendix. Data that are not presented in the Article or appendix are available upon reasonable request to CIDACS.

Any person who wishes to receive authorisation must: (i) be affiliated to CIDACS or be accepted as collaborators; (ii) present a detailed research project together with approval by an appropriate Brazilian institutional research ethical committee; (iii) provide a clear data plan restricted to the objectives of the proposed study and a summary of the analyses plan intended to guide the linkage and or data extraction of the relevant set of records and variables; (iv) sign terms of responsibility regarding the access and use of data; and (v) perform the analyses of datasets provided using the CIDACS data environment, a safe and secure infrastructure that provides remote access to de-identified or anonymized datasets and analysis tools.

## Editor note

The Lancet Group takes a neutral position with respect to territorial claims in published maps and institutional affiliations.

## Declaration of interests

MLB is a Brazilian National Research Council research fellow. ESP and JMP acknowledge funding from the Wellcome Trust (225925/Z/22/Z to ESP, 305644/Z/23/Z to JMP). TC-S acknowledges funding from the Royal Society (NIF∖R1∖231435). All other authors declare no competing interests.

## Appendix A Supplementary data

Supplementary R2
