# Supplementary material for: Perinatal health outcomes of offspring of internal migrant women according to human development index: a registry-based cohort study of over 10 million live births from Brazil
Source: Lancet Reg Health Am. 2025 Feb 12;43:101020. doi: 10.1016/j.lana.2025.101020 (PMC11870261; doi:10.1016/j.lana.2025.101020)
Supplement: Supplementary R2 [file mmc1.docx]

Table of Contents

[Supplementary Table 1. Marginal Risk Ratio (RR) for the association between migration and perinatal outcomes when mothers migrate to municipalities with equal/higher or lower human development index (HDI). 1](#_Toc188618113)

[Supplementary Table 2. Sociodemographic and economic characteristics of live births of internal migrant women moving to equal/higher HDI or lower HDI and non-migrant women in the sibling design. 1](#_Toc188618114)

[Supplementary Table 3. Conditional Odds Ratio (OR) for the association between migration and perinatal outcomes when mothers migrate to municipalities with higher/equal or lower human development index (HDI) in the sibling design. 3](#_Toc188618115)

[Supplementary Table 4. Marginal Risk Ratio (RR) for the association between migration and perinatal outcomes when mothers migrate to municipalities with equal/higher or lower human development index (HDI) using multiple imputation instead missing indicator. 3](#_Toc188618116)

[Supplementary Table 5. Main causes of congenital malformations by ICD-10 among children according to migration status. The live birth can be categorised in more than one congenital malformation 4](#_Toc188618117)

[Supplementary Table 6. Marginal Risk Ratio for the association between migration and perinatal outcomes when mothers migrate to municipalities with higher or equal/lower human development index (HDI) adjusted for HDI of the municipality of arrival. 4](#_Toc188618118)

[Supplementary Table 7. Marginal Risk Ratio for the association between migration and perinatal outcomes stratifying by human development index (HDI) and standardised number of nurses per 1,000 inhabitants for the year of birth 5](#_Toc188618119)

[Supplementary Figure 1A: Brazil’s map showing the geographic regions and States in each region. 6](#_Toc188618120)

[Supplementary Figure 1B: Brazil’s map showing the human development index (HDI) of each municipality (5570) 7](#_Toc188618121)

[Supplementary Figure 2: Histogram of the differences in human development index (HDI) of the municipalities of registration in the CadUnico and municipality of arrival (live birth system) in those classified as migrant. 8](#_Toc188618122)

[Supplementary Figure 3: Directed acyclic graphic showing the assumed relationship between the variables in the outcome model. Outcomes evaluated: (i) timely initiation of antenatal care (at least one antenatal appointment within the first trimester), (ii) preterm (gestational age <37 weeks), (iii) low birth weight (LBW; birth weight <2500 g), (v) small for gestational age (SGA; weight below the 10th percentile for gestational age and sex), (vi) low Apgar score (Apgar score below 7 at 5 minutes after birth), (vii) congenital anomaly at birth, and (viii) neonatal mortality (death up to 28 days of life). 9](#_Toc188618123)

[Supplementary Figure 4: Love plots representing the effect of the weighting in terms of standardized mean differences (SMD) among the weighted variables. CadU = CadUnico. Higher HDI = Equal/Higher HDI 9](#_Toc188618124)

[Supplementary Figure 5: Risk Ratio of perinatal outcomes of women according to migration by the difference in the Human Development Index (HDI) between municipality of origin and arrival. Positive values refer to migration to higher HDI, whilst negative values refer to migration to lower HDIs. 10](#_Toc188618125)

[STROBE Statement—Checklist of items that should be included in reports of cohort studies 11](#_Toc188618126)

# **Supplementary Table 1.** Marginal Risk Ratio (RR) for the association between migration and perinatal outcomes when mothers migrate to municipalities with equal/higher or lower human development index (HDI).

|  |  | **Equal/Higher HDI municipalities** | |  | **Lower HDI municipalities** | |
| --- | --- | --- | --- | --- | --- | --- |
| **Outcome** | **Crude Risk Ratio** | **IPW adjustment**  **Risk Ratio (95%CI)** | **IPW + time varying variables**  **Risk Ratio (95%CI)** | **Crude Risk Ratio** | **IPW adjustment**  **Risk Ratio (95%CI)** | **IPW + time varying variables**  **Risk Ratio (95%CI)** |
| **Congenital abnormalities** | 1.25 (1.21 to 1.29) | 1.41 (1.37 to 1.46) | 1.14 (1.1 to 1.18) | 0.93 (0.87 to 0.99) | 0.85 (0.79 to 0.91) | 0.97 (0.9 to 1.03) |
| **≤0.1** | 1.04 (0.99 to 1.10) | 1.11 (1.05 to 1.17) | 1.02 (0.97 to 1.08) | 0.94 (0.87 to 1.01) | 0.89 (0.82 to 0.96) | 0.98 (0.9 to 1.06) |
| **>0.1** | 1.36 (1.31 to 1.41) | 1.59 (1.53 to 1.66) | 1.13 (1.07 to 1.19) | 0.89 (0.79 to 1.01) | 0.75 (0.66 to 0.85) | 0.91 (0.8 to 1.04) |
| **Delayed antenatal care (Start >3rd month)** | 0.90 (0.90 to 0.91) | 0.88 (0.87 to 0.88) | 1.00 (0.99 to 1.01) | 1.06 (1.05 to 1.07) | 1.13 (1.12 to 1.14) | 1.01 (1.00 to 1.02) |
| **≤0.1** | 0.95 (0.94 to 0.96) | 0.97 (0.96 to 0.98) | 1.02 (1.01 to 1.03) | 1.02 (1.01 to 1.03) | 1.08 (1.07 to 1.1) | 1.01 (1.00 to 1.02) |
| **>0.1** | 0.88 (0.87 to 0.89) | 0.83 (0.82 to 0.84) | 0.99 (0.98 to 1.00) | 1.17 (1.15 to 1.19) | 1.24 (1.22 to 1.26) | 1.01 (0.99 to 1.02) |
| **Neonatal Death (≤28 days)** | 0.91 (0.87 to 0.94) | 0.87 (0.84 to 0.9) | 0.99 (0.95 to 1.03) | 1.06 (0.99 to 1.13) | 1.10 (1.02 to 1.17) | 1.07 (1.00 to 1.15) |
| **≤0.1** | 0.96 (0.90 to 1.02) | 0.96 (0.9 to 1.02) | 1.01 (0.95 to 1.08) | 1.05 (0.97 to 1.14) | 1.09 (1.00 to 1.18) | 1.07 (0.98 to 1.16) |
| **>0.1** | 0.88 (0.84 to 0.92) | 0.82 (0.78 to 0.87) | 0.99 (0.93 to 1.05) | 1.09 (0.96 to 1.23) | 1.12 (0.99 to 1.27) | 1.10 (0.98 to 1.25) |
| **Low Apgar 5’ (<7)** | 0.96 (0.93 to 0.98) | 0.87 (0.85 to 0.9) | 0.99 (0.96 to 1.02) | 1.02 (0.96 to 1.07) | 1.09 (1.03 to 1.15) | 1.03 (0.98 to 1.09) |
| **≤0.1** | 0.94 (0.90 to 0.99) | 0.98 (0.94 to 1.03) | 0.98 (0.94 to 1.03) | 0.98 (0.92 to 1.05) | 1.04 (0.97 to 1.11) | 1.01 (0.94 to 1.08) |
| **>0.1** | 0.96 (0.93 to 1.00) | 0.83 (0.80 to 0.86) | 0.96 (0.92 to 1.01) | 1.10 (1.00 to 1.21) | 1.21 (1.09 to 1.33) | 1.12 (1.01 to 1.23) |
| **Low birth weight (<2500g)** | 0.95 (0.94 to 0.96) | 1.06 (1.05 to 1.07) | 0.94 (0.93 to 0.95) | 0.94 (0.92 to 0.96) | 0.90 (0.88 to 0.92) | 0.94 (0.92 to 0.96) |
| **≤0.1** | 0.96 (0.94 to 0.98) | 0.99 (0.98 to 1.01) | 0.95 (0.93 to 0.97) | 0.94 (0.92 to 0.97) | 0.92 (0.90 to 0.95) | 0.94 (0.92 to 0.96) |
| **>0.1** | 0.94 (0.93 to 0.96) | 1.10 (1.08 to 1.11) | 0.95 (0.93 to 0.97) | 0.93 (0.90 to 0.97) | 0.85 (0.82 to 0.89) | 0.94 (0.9 to 0.98) |
| **Preterm (<37 weeks)** | 0.89 (0.89 to 0.90) | 0.91 (0.9 to 0.92) | 0.94 (0.93 to 0.95) | 1.01 (0.99 to 1.02) | 1.02 (1.00 to 1.03) | 1.00 (0.98 to 1.01) |
| **≤0.1** | 0.95 (0.94 to 0.97) | 0.97 (0.95 to 0.98) | 0.97 (0.96 to 0.99) | 1.00 (0.98 to 1.02) | 1.01 (0.99 to 1.03) | 0.99 (0.97 to 1.01) |
| **>0.1** | 0.86 (0.85 to 0.87) | 0.88 (0.87 to 0.89) | 0.94 (0.92 to 0.95) | 1.03 (1.00 to 1.06) | 1.04 (1.01 to 1.07) | 1.01 (0.98 to 1.04) |
| **Small for gestational age (<10th%)** | 0.94 (0.93 to 0.95) | 0.93 (0.92 to 0.94) | 0.92 (0.91 to 0.93) | 1.00 (0.99 to 1.02) | 1.03 (1.01 to 1.05) | 1.00 (0.99 to 1.02) |
| **≤0.1** | 0.95 (0.93 to 0.96) | 0.96 (0.94 to 0.97) | 0.96 (0.94 to 0.98) | 0.97 (0.95 to 0.99) | 1.00 (0.98 to 1.02) | 0.98 (0.96 to 1.01) |
| **>0.1** | 0.94 (0.93 to 0.95) | 0.91 (0.90 to 0.92) | 0.90 (0.89 to 0.92) | 1.08 (1.05 to 1.12) | 1.09 (1.05 to 1.13) | 1.06 (1.03 to 1.10) |

IPW model adjusted for: date of registration in CadUnico, state of residency, state of cohort registry different from birth, location of household (rural or urban area), material of household, water system, waste disposal/garbage collection, HDI of the municipality, age, education level, race/ethnicity.

Time varying variables: age of the mother, state of residence, number of antenatal visits, education level, year of conception, parity, receipt of conditional cash transfer benefit, marital status, and previous foetal loss

HDI: Human Development Index | IPW: Inverse Probability Weigthing

# **Supplementary Table 2.** Sociodemographic and economic characteristics of live births of internal migrant women moving to equal/higher HDI or lower HDI and non-migrant women in the sibling design.

| **Characteristic** | **Higher/Equal HDI**  **N = 104,187** | **Lower HDI**  **N = 33,285** | **Non-migrants**  **N = 2,846,811** | **Overall**  **N = 2,984,283** |
| --- | --- | --- | --- | --- |
| **No. women** | 70,487 | 23,798 | 1,361,874 | 1,456,159 |
| ***Mother’s characteristics at CadU enter*** | |  |  |  |
| Water system |  |  |  |  |
| Public system | 55,992 (58.6%) | 23,324 (75.9%) | 1,821,053 (67.3%) | 1,900,369 (67.1%) |
| Water well | 28,916 (30.2%) | 5,361 (17.5%) | 626,337 (23.1%) | 660,614 (23.3%) |
| Other | 10,683 (11.2%) | 2,035 (6.6%) | 258,599 (9.6%) | 271,317 (9.6%) |
| Missing | 8,596 | 2,565 | 140,822 | 151,983 |
| **Material of the household** |  |  |  |  |
| Masonry / brick | 57,304 (59.9%) | 22,260 (72.5%) | 1,828,292 (67.6%) | 1,907,856 (67.4%) |
| Coated Taipa | 5,939 (6.2%) | 799 (2.6%) | 102,246 (3.8%) | 108,984 (3.8%) |
| Uncoated Taipa | 6,094 (6.4%) | 742 (2.4%) | 109,843 (4.1%) | 116,679 (4.1%) |
| Wood | 13,272 (13.9%) | 5,268 (17.1%) | 457,863 (16.9%) | 476,403 (16.8%) |
| Other | 12,985 (13.6%) | 1,651 (5.4%) | 207,733 (7.7%) | 222,369 (7.9%) |
| Missing | 8,593 | 2,565 | 140,834 | 151,992 |
| **Location of household** |  |  |  |  |
| Urban | 60,964 (63.3%) | 26,190 (84.6%) | 2,056,294 (75.2%) | 2,143,448 (74.9%) |
| Rural | 35,412 (36.7%) | 4,777 (15.4%) | 678,976 (24.8%) | 719,165 (25.1%) |
| Missing | 7,811 | 2,318 | 111,541 | 121,670 |
| **Household type** |  |  |  |  |
| Permanent (independent of ownership) | 91,867 (97.1%) | 28,522 (94.6%) | 2,547,737 (95.8%) | 2,668,126 (95.8%) |
| Improvised (e.g., shacks) | 420 (0.4%) | 143 (0.5%) | 18,100 (0.7%) | 18,663 (0.7%) |
| Collective (household occupied by multiple families) | 46 (0.0%) | 29 (0.1%) | 2,373 (0.1%) | 2,448 (0.1%) |
| Other | 2,251 (2.4%) | 1,448 (4.8%) | 91,167 (3.4%) | 94,866 (3.4%) |
| Missing | 9,603 | 3,143 | 187,434 | 200,180 |
| **Waste disposal/ garbage collection** | |  |  |  |
| Collected direct/indirectly | 54,906 (57.4%) | 25,095 (81.7%) | 1,925,435 (71.2%) | 2,005,436 (70.8%) |
| Burned or buried | 25,511 (26.7%) | 3,785 (12.3%) | 529,423 (19.6%) | 558,719 (19.7%) |
| Open air dump | 14,011 (14.7%) | 1,388 (4.5%) | 213,774 (7.9%) | 229,173 (8.1%) |
| Other | 1,169 (1.2%) | 455 (1.5%) | 37,341 (1.4%) | 38,965 (1.4%) |
| Missing | 8,590 | 2,562 | 140,838 | 151,990 |
| **Age – at CadU enter** | 15 (12, 19) | 15 (12, 21) | 15 (12, 20) | 15 (12, 20) |
| **Education level** |  |  |  |  |
| No school | 3,987 (4.8%) | 1,458 (5.4%) | 133,254 (5.5%) | 138,699 (5.5%) |
| Nursery | 736 (0.9%) | 325 (1.2%) | 26,377 (1.1%) | 27,438 (1.1%) |
| Infant School (learning to read/write) | 898 (1.1%) | 245 (0.9%) | 28,332 (1.2%) | 29,475 (1.2%) |
| Middle school 1 | 32,351 (39.0%) | 10,382 (38.5%) | 929,805 (38.5%) | 972,538 (38.5%) |
| Middle school 2 | 34,550 (41.7%) | 10,856 (40.3%) | 932,347 (38.6%) | 977,753 (38.7%) |
| High School | 10,116 (12.2%) | 3,540 (13.1%) | 353,524 (14.6%) | 367,180 (14.5%) |
| Higher education | 228 (0.3%) | 132 (0.5%) | 11,867 (0.5%) | 12,227 (0.5%) |
| Missing | 21,321 | 6,347 | 431,305 | 458,973 |
| **Race/ethnicity** |  |  |  |  |
| White | 20,620 (22.0%) | 9,368 (30.6%) | 715,219 (27.0%) | 745,207 (26.9%) |
| Black | 6,573 (7.0%) | 2,405 (7.9%) | 230,976 (8.7%) | 239,954 (8.7%) |
| Mixed | 65,542 (70.0%) | 18,507 (60.5%) | 1,651,313 (62.3%) | 1,735,362 (62.6%) |
| Asian | 442 (0.5%) | 92 (0.3%) | 9,389 (0.4%) | 9,923 (0.4%) |
| Indigenous | 488 (0.5%) | 218 (0.7%) | 42,268 (1.6%) | 42,974 (1.5%) |
| Missing | 10,522 | 2,695 | 197,646 | 210,863 |
| **Municipality HDI – CadU enter** | 0.61 (0.57, 0.68) | 0.75 (0.70, 0.79) | 0.70 (0.61, 0.76) | 0.70 (0.61, 0.75) |
| **State of CadU enter different from birth state** | 20,243 (19.9%) | 12,871 (40.0%) | 249,949 (9.4%) | 283,063 (10.2%) |
| Missing | 2,414 | 1,094 | 201,258 | 204,766 |
| **CadU – Register geographical region** | |  |  |  |
| 1 | 13,561 (13.0%) | 4,537 (13.6%) | 432,467 (15.2%) | 450,565 (15.1%) |
| 2 | 65,515 (62.9%) | 8,350 (25.1%) | 1,058,812 (37.2%) | 1,132,677 (38.0%) |
| 3 | 11,772 (11.3%) | 11,154 (33.5%) | 838,816 (29.5%) | 861,742 (28.9%) |
| 4 | 7,839 (7.5%) | 3,929 (11.8%) | 304,005 (10.7%) | 315,773 (10.6%) |
| 5 | 5,500 (5.3%) | 5,315 (16.0%) | 212,711 (7.5%) | 223,526 (7.5%) |
| ***Mother’s characteristics at Pregnancy*** | |  |  |  |
| **Age at Pregnancy** |  |  |  |  |
| 10-17 | 9,347 (9.0%) | 3,900 (11.7%) | 337,474 (11.9%) | 350,721 (11.8%) |
| 18-24 | 53,579 (51.4%) | 15,773 (47.4%) | 1,379,566 (48.5%) | 1,448,918 (48.6%) |
| 25-29 | 26,380 (25.3%) | 7,466 (22.4%) | 637,851 (22.4%) | 671,697 (22.5%) |
| 30-34 | 11,075 (10.6%) | 4,198 (12.6%) | 333,081 (11.7%) | 348,354 (11.7%) |
| 35-49 | 3,806 (3.7%) | 1,948 (5.9%) | 158,839 (5.6%) | 164,593 (5.5%) |
| **Number of prenatal appointments** | |  |  |  |
| None | 2,474 (2.4%) | 857 (2.6%) | 76,838 (2.7%) | 80,169 (2.7%) |
| 1 to 3 | 11,815 (11.4%) | 4,409 (13.3%) | 358,484 (12.7%) | 374,708 (12.7%) |
| 4 to 6 | 33,408 (32.3%) | 11,949 (36.2%) | 976,914 (34.6%) | 1,022,271 (34.5%) |
| ≥7 | 55,621 (53.8%) | 15,820 (47.9%) | 1,412,320 (50.0%) | 1,483,761 (50.1%) |
| Missing | 869 | 250 | 22,255 | 23,374 |
| **Pregnancy – Geographical region** | |  |  |  |
| North | 11,905 (11.4%) | 5,332 (16.0%) | 432,467 (15.2%) | 449,704 (15.1%) |
| Northeast | 11,523 (11.1%) | 12,587 (37.8%) | 1,058,812 (37.2%) | 1,082,922 (36.3%) |
| Southeast | 48,221 (46.3%) | 6,180 (18.6%) | 838,816 (29.5%) | 893,217 (29.9%) |
| South | 10,057 (9.7%) | 3,705 (11.1%) | 304,005 (10.7%) | 317,767 (10.6%) |
| Central west | 22,481 (21.6%) | 5,481 (16.5%) | 212,711 (7.5%) | 240,673 (8.1%) |
| **Education level at birth** |  |  |  |  |
| None | 585 (0.6%) | 408 (1.2%) | 28,546 (1.0%) | 29,539 (1.0%) |
| 1 to 3 | 4,405 (4.3%) | 1,932 (5.9%) | 150,175 (5.4%) | 156,512 (5.3%) |
| 4 to 7 | 32,652 (31.7%) | 11,263 (34.4%) | 914,831 (32.7%) | 958,746 (32.6%) |
| 8 to 11 | 61,448 (59.7%) | 17,787 (54.4%) | 1,606,805 (57.4%) | 1,686,040 (57.4%) |
| ≥12 | 3,888 (3.8%) | 1,308 (4.0%) | 100,455 (3.6%) | 105,651 (3.6%) |
| Missing | 1,209 | 587 | 45,999 | 47,795 |
| **Pregnancy Year** |  |  |  |  |
| 2010 | 5,948 (5.7%) | 2,220 (6.7%) | 180,814 (6.4%) | 188,982 (6.3%) |
| 2011 | 14,474 (13.9%) | 4,345 (13.1%) | 355,412 (12.5%) | 374,231 (12.5%) |
| 2012 | 17,810 (17.1%) | 5,441 (16.3%) | 401,298 (14.1%) | 424,549 (14.2%) |
| 2013 | 20,782 (19.9%) | 6,801 (20.4%) | 418,547 (14.7%) | 446,130 (14.9%) |
| 2014 | 21,334 (20.5%) | 6,640 (19.9%) | 434,729 (15.3%) | 462,703 (15.5%) |
| 2015 | 10,211 (9.8%) | 3,253 (9.8%) | 350,918 (12.3%) | 364,382 (12.2%) |
| 2016 | 5,840 (5.6%) | 1,897 (5.7%) | 320,551 (11.3%) | 328,288 (11.0%) |
| 2017 | 7,123 (6.8%) | 2,452 (7.4%) | 352,356 (12.4%) | 361,931 (12.1%) |
| 2018 | 665 (0.6%) | 236 (0.7%) | 32,186 (1.1%) | 33,087 (1.1%) |
| **Nulliparous** | 22,119 (21.8%) | 6,155 (19.1%) | 545,688 (19.8%) | 573,962 (19.9%) |
| Missing | 2,833 | 1,011 | 90,934 | 94,778 |
| **Civil status** |  |  |  |  |
| Single | 54,247 (52.5%) | 15,051 (45.8%) | 1,450,885 (51.6%) | 1,520,183 (51.6%) |
| Common Law – Marriage | 34,929 (33.8%) | 12,958 (39.4%) | 894,453 (31.8%) | 942,340 (32.0%) |
| Married | 13,379 (13.0%) | 4,572 (13.9%) | 444,909 (15.8%) | 462,860 (15.7%) |
| Divorced | 579 (0.6%) | 223 (0.7%) | 17,734 (0.6%) | 18,536 (0.6%) |
| Widowed | 124 (0.1%) | 48 (0.1%) | 4,362 (0.2%) | 4,534 (0.2%) |
| Missing | 929 | 433 | 34,468 | 35,830 |
| **Previous stillbirth** | 19,638 (20.2%) | 6,798 (22.3%) | 528,080 (20.2%) | 554,516 (20.3%) |
| Missing | 6,880 | 2,755 | 237,792 | 247,427 |
| **Municipality HDI – Pregnancy** | 0.76 (0.72, 0.81) | 0.67 (0.60, 0.72) | 0.71 (0.62, 0.76) | 0.71 (0.62, 0.76) |
| **Ever received conditional cash transfer (yes)** | 99,266 (95.3%) | 31,471 (94.6%) | 2,680,804 (94.2%) | 2,811,541 (94.2%) |
| **Low birth weight (<2500g)** | 7,172 (6.9%) | 204,296 (7.2%) | 2,270 (6.8%) | 213,738 (7.2%) |
| **Preterm (<37 weeks)** | 11,238 (10.8%) | 336,447 (11.8%) | 3,967 (11.9%) | 351,652 (11.8%) |
| **Low APGAR 5’ (<7)** | 1,264 (1.2%) | 34,429 (1.3%) | 387 (1.2%) | 36,080 (1.3%) |
| Missing | 1,722 | 114,803 | 1,289 | 117,814 |
| **Size for gestational age** |  |  |  |  |
| Adequate | 78,623 (75.5%) | 2,112,761 (74.2%) | 24,543 (73.7%) | 2,215,927 (74.3%) |
| Small | 7,980 (7.7%) | 236,244 (8.3%) | 2,777 (8.3%) | 247,001 (8.3%) |
| Large | 17,584 (16.9%) | 497,806 (17.5%) | 5,965 (17.9%) | 521,355 (17.5%) |
| **Delayed antenatal care (Start >3rd month)** | 30,476 (31.6%) | 878,520 (33.4%) | 10,587 (34.4%) | 919,583 (33.3%) |
| Missing | 7,881 | 215,040 | 2,501 | 225,422 |
| **Congenital abnormalities** | 1,048 (1.0%) | 22,984 (0.8%) | 241 (0.7%) | 24,273 (0.8%) |
| Missing | 3,177 | 68,401 | 1,122 | 72,700 |
| **Neonatal Death (≤28 days)** | 1,020 (1.0%) | 28,717 (1.0%) | 327 (1.0%) | 30,064 (1.0%) |

# **Supplementary Table 3.** Conditional Odds Ratio (OR) for the association between migration and perinatal outcomes when mothers migrate to municipalities with higher/equal or lower human development index (HDI) in the sibling design.

|  | **Equal/Higher HDI municipalities** | | **Lower HDI municipalities** | |
| --- | --- | --- | --- | --- |
| **Outcome** | **Crude OR (95%CI)** | **Adjusted OR (95%CI)** | **Crude OR (95%CI)** | **Adjusted OR (95%CI)** |
| **Congenital abnormalities** | 1.36 (1.18 to 1.58) | 1.45 (1.25 to 1.68) | 0.97 (0.75 to 1.24) | 1.04 (0.80 to 1.34) |
| **≤0.1** |  | 1.25 (1.00 to 1.56) | | 1.04 (0.77 to 1.40) |
| **>0.1** |  | 1.60 (1.32 to 1.92) | | 0.99 (0.63 to 1.55) |
| **Delayed antenatal care (Start >3rd month)** | 0.9 (0.87 to 0.93) | 0.87 (0.84 to 0.90) | 1.08 (1.02 to 1.14) | 1.05 (1.00 to 1.11) |
| **≤0.1** |  | 0.96 (0.91 to 1.01) | | 0.99 (0.93 to 1.06) |
| **>0.1** |  | 0.82 (0.79 to 0.85) | | 1.25 (1.13 to 1.37) |
| **Neonatal Death (≤28 days)** | 0.77 (0.67 to 0.89) | 0.83 (0.71 to 0.97) | 1.19 (0.94 to 1.5) | 1.33 (1.03 to 1.72) |
| **≤0.1** |  | 1.01 (0.79 to 1.28) | | 1.51 (1.11 to 2.03) |
| **>0.1** |  | 0.73 (0.60 to 0.89) | | 1.05 (0.67 to 1.65) |
| **Low Apgar 5’ (<7)** | 0.88 (0.78 to 1) | 0.93 (0.81 to 1.06) | 1.09 (0.88 to 1.35) | 1.12 (0.90 to 1.39) |
| **≤0.1** |  | 1.02 (0.84 to 1.24) | | 1.11 (0.86 to 1.42) |
| **>0.1** |  | 0.87 (0.74 to 1.02) | | 1.18 (0.78 to 1.77) |
| **Low birth weight (<2500g)** | 1.08 (1.02 to 1.15) | 1.09 (1.03 to 1.16) | 0.97 (0.88 to 1.06) | 0.97 (0.88 to 1.07) |
| **≤0.1** |  | 1.06 (0.97 to 1.16) | | 0.92 (0.83 to 1.03) |
| **>0.1** |  | 1.12 (1.03 to 1.21) | | 1.08 (0.92 to 1.28) |
| **Preterm (<37 weeks)** | 0.87 (0.83 to 0.91) | 0.88 (0.84 to 0.92) | 1.03 (0.96 to 1.1) | 1.04 (0.97 to 1.12) |
| **≤0.1** |  | 0.97 (0.91 to 1.03) | | 0.98 (0.91 to 1.07) |
| **>0.1** |  | 0.82 (0.77 to 0.87) | | 1.23 (1.08 to 1.39) |
| **Small for gestational age (<10th%)** | 0.91 (0.86 to 0.96) | 0.92 (0.87 to 0.97) | 0.99 (0.91 to 1.08) | 1.01 (0.93 to 1.10) |
| **≤0.1** |  | 0.94 (0.86 to 1.01) |  | 0.96 (0.87 to 1.06) |
| **>0.1** |  | 0.90 (0.84 to 0.96) |  | 1.13 (0.98 to 1.31) |

*Adjusted for mother’s age, year of conception, education level, order of birth and marital status.

# **Supplementary Table 4.** Marginal Risk Ratio (RR) for the association between migration and perinatal outcomes when mothers migrate to municipalities with equal/higher or lower human development index (HDI) using multiple imputation instead missing indicator.

|  | **Higher/Equal HDI municipalities** | | **Lower HDI municipalities** | |
| --- | --- | --- | --- | --- |
| **Outcome** | **IPW adjustment**  **Risk Ratio (95% CI)** | **IPW + time varying variables**  **Risk Ratio (95% CI)** | **IPW adjustment**  **Risk Ratio (95% CI)** | **IPW + time varying variables**  **Risk Ratio (95% CI)** |
| **Congenital abnormalities** | 1.39 (1.35 to 1.44) | 1.14 (1.10 to 1.18) | 0.85 (0.79 to 0.91) | 0.97 (0.90 to 1.03) |
| **Delayed antenatal care (Start >3rd month)** | 0.88 (0.87 to 0.88) | 1.00 (0.99 to 1.00) | 1.11 (1.10 to 1.13) | 1.01 (1.00 to 1.02) |
| **Neonatal Death**  **(≤28 days)** | 0.86 (0.83 to 0.90) | 0.99 (0.95 to 1.03) | 1.09 (1.02 to 1.17) | 1.07 (1.00 to 1.14) |
| **Low Apgar 5’**  **(<7)** | 0.87 (0.84 to 0.89) | 0.99 (0.96 to 1.02) | 1.08 (1.02 to 1.14) | 1.03 (0.97 to 1.08) |
| **Low birth weight (<2500g)** | 1.06 (1.04 to 1.07) | 0.94 (0.93 to 0.95) | 0.89 (0.88 to 0.91) | 0.94 (0.92 to 0.96) |
| **Preterm**  **(<37 weeks)** | 0.91 (0.90 to 0.92) | 0.94 (0.93 to 0.95) | 1.01 (0.99 to 1.03) | 1.00 (0.98 to 1.01) |
| **Small for gestational age (<10th%)** | 0.93 (0.92 to 0.94) | 0.92 (0.91 to 0.94) | 1.02 (1.00 to 1.04) | 1.00 (0.98 to 1.02) |

IPW model adjusted for: date of registration in CadUnico, state of residency, state of cohort registry different from birth, location of household (rural or urban area), material of household, water system, waste disposal/garbage collection, HDI of the municipality, age, education level, race/ethnicity.

Time varying variables: age of the mother, state of residence, number of antenatal visits, education level, year of conception, parity, receipt of conditional cash transfer benefit, marital status, and previous foetal loss

HDI: Human Development Index

IPW: Inverse Probability Weighting

# **Supplementary Table 5.** Main causes of congenital malformations by ICD-10 among children according to migration status. The live birth can be categorised in more than one congenital malformation

| **ICD-10 Group** | **Equal/Higher HDI municipalities**  **N = 458,002** | | **Non-migrants**  **N = 9,603,945** | **Lower HDI municipalities**  **N = 122,074** | **Overall**  **N = 10,184,021** |
| --- | --- | --- | --- | --- | --- |
| **Congenital malformations of the nervous system** | 512 (0.11%) | 10,601 (0.11%) | | 133 (0.11%) | 11,246 (0.11%) |
| **Congenital malformations of eye, ear, face and neck** | 383 (0.08%) | 5,596 (0.06%) | | 65 (0.05%) | 6,044 (0.06%) |
| **Congenital malformations of the circulatory system** | 598 (0.13%) | 5,286 (0.06%) | | 48 (0.04%) | 5,932 (0.06%) |
| **Congenital malformations of the respiratory system** | 513 (0.11%) | 9,031 (0.09%) | | 113 (0.09%) | 9,657 (0.09%) |
| **Cleft lip and cleft palate** | 513 (0.11%) | 9,031 (0.09%) | | 113 (0.09%) | 9,657 (0.09%) |
| **Other congenital malformations of the digestive system** | 606 (0.13%) | 10,703 (0.11%) | | 136 (0.11%) | 11,445 (0.11%) |
| **Congenital malformations of genital organs** | 418 (0.09%) | 6,618 (0.07%) | | 74 (0.06%) | 7,110 (0.07%) |
| **Congenital malformations of the urinary system** | 1,446 (0.32%) | 26,277 (0.27%) | | 311 (0.25%) | 28,034 (0.28%) |
| **Congenital malformations and deformations of the musculoskeletal system** | 2,073 (0.45%) | 37,146 (0.39%) | | 452 (0.37%) | 39,671 (0.39%) |
| **Other congenital malformations** | 278 (0.06%) | 4,824 (0.05%) | | 55 (0.05%) | 5,157 (0.05%) |
| **Chromosomal abnormalities, not elsewhere classified** | 160 (0.03%) | 3,197 (0.03%) | | 51 (0.04%) | 3,408 (0.03%) |

# **Supplementary Table 6.** Marginal Risk Ratio for the association between migration and perinatal outcomes when mothers migrate to municipalities with higher or equal/lower human development index (HDI) adjusted for HDI of the municipality of arrival.

| **Outcome** | **Equal/Higher HDI municipalities** | **Lower HDI municipalities** |
| --- | --- | --- |
|  | **Risk Ratio (95% CI)** | **Risk Ratio (95% CI)** |
| **Congenital abnormalities** | 1.09 (1.06 to 1.13) | 1.06 (0.99 to 1.14) |
| **Delayed antenatal care (Start >3rd month)** | 1.05 (1.02 to 1.09) | 0.98 (0.92 to 1.03) |
| **Neonatal Death (≤28 days)** | 1.03 (0.99 to 1.08) | 1.02 (0.95 to 1.09) |
| **Low Apgar 5’ (<7)** | 1.00 (1.00 to 1.01) | 1.02 (1.01 to 1.03) |
| **Low birth weight (<2500g)** | 0.91 (0.90 to 0.92) | 0.98 (0.96 to 1.00) |
| **Preterm birth (<37 weeks)** | 0.94 (0.93 to 0.95) | 0.99 (0.97 to 1.00) |
| **Small for gestational age (<10th%)** | 0.94 (0.93 to 0.95) | 0.99 (0.97 to 1.00) |

*Adjusted through IPW + Time varying variables (age of the mother, state of residence, number of antenatal visits, education level, year of conception, parity, receipt of conditional cash transfer benefit, marital status, and previous foetal loss) + HDI of the municipality of arrival

# **Supplementary Table 7.** Marginal Risk Ratio for the association between migration and perinatal outcomes stratifying by human development index (HDI) and standardised number of nurses per 1,000 inhabitants for the year of birth

| **Outcomes** | **To equal/higher HDI municipality and equal/higher no. nurse per inhabitants (N=336,429)** | | | **To equal/higher HDI municipality and lower no. nurse per inhabitants (N=121,573)** | |
| --- | --- | --- | --- | --- | --- |
|  | **IPW adjustment** | **IPW + time varying variables** | **IPW adjustment** | | **IPW + time varying variables** |
| **Congenital abnormalities** | 1.51 (1.46 to 1.57) | 1.07 (1.01 to 1.13) | | 1.15 (1.08 to 1.22) | 1.06 (0.96 to 1.16) |
| **Delayed antenatal care (Start >3rd month)** | 0.88 (0.87 to 0.88) | 1.01 (1.00 to 1.02) | | 0.89 (0.88 to 0.9) | 0.99 (0.98 to 1.01) |
| **Neonatal Death (≤28 days)** | 0.86 (0.82 to 0.90) | 1.01 (0.95 to 1.08) | | 0.90 (0.84 to 0.97) | 0.94 (0.85 to 1.05) |
| **Low Apgar 5’ (<7)** | 0.85 (0.82 to 0.88) | 0.93 (0.89 to 0.98) | | 0.93 (0.88 to 0.98) | 0.98 (0.90 to 1.05) |
| **Low birth weight (<2500g)** | 1.07 (1.06 to 1.09) | 1.00 (0.98 to 1.02) | | 1.02 (1.00 to 1.04) | 0.95 (0.92 to 0.98) |
| **Preterm (<37 weeks)** | 0.91 (0.90 to 0.92) | 0.97 (0.95 to 0.99) | | 0.92 (0.90 to 0.94) | 0.95 (0.93 to 0.98) |
| **Small for gestational age (<10th%)** | 0.92 (0.91 to 0.93) | 0.92 (0.91 to 0.94) | | 0.94 (0.93 to 0.96) | 0.95 (0.93 to 0.98) |
| **Outcomes** | **To lower HDI municipality and equal/higher no. nurse per inhabitants (N=36,713)** | | | **To lower HDI municipality and lower no. nurse per inhabitants (N=85,360)** | |
|  | **IPW adjustment**  **Risk Ratio (95% CI)** | **IPW + time varying variables**  **Risk Ratio (95% CI)** | | **IPW adjustment**  **Risk Ratio (95% CI)** | **IPW + time varying variables**  **Risk Ratio (95% CI)** |
| **Congenital abnormalities** | 0.90 (0.80 to 1.01) | 1.02 (0.90 to 1.17) | | 0.82 (0.76 to 0.89) | 0.96 (0.88 to 1.06) |
| **Delayed antenatal care (Start >3rd month)** | 1.13 (1.11 to 1.15) | 1.00 (0.98 to 1.02) | | 1.13 (1.12 to 1.14) | 1.01 (1.00 to 1.03) |
| **Neonatal Death (≤28 days)** | 1.05 (0.93 to 1.19) | 0.98 (0.85 to 1.12) | | 1.11 (1.03 to 1.21) | 1.10 (1.01 to 1.21) |
| **Low Apgar 5’ (<7)** | 1.11 (1.01 to 1.22) | 1.06 (0.95 to 1.18) | | 1.08 (1.01 to 1.15) | 1.05 (0.98 to 1.13) |
| **Low birth weight (<2500g)** | 0.92 (0.89 to 0.96) | 0.98 (0.93 to 1.02) | | 0.89 (0.87 to 0.91) | 0.93 (0.91 to 0.96) |
| **Preterm (<37 weeks)** | 1.03 (1.00 to 1.06) | 1.02 (0.99 to 1.06) | | 1.01 (0.99 to 1.03) | 0.99 (0.97 to 1.01) |
| **Small for gestational age (<10th%)** | 1.04 (1.01 to 1.08) | 1.04 (1.00 to 1.08) | | 1.02 (0.99 to 1.04) | 1.00 (0.98 to 1.03) |

IPW model adjusted for: date of registration in CadUnico, state of residency, state of cohort registry different from birth, location of household (rural or urban area), material of household, water system, waste disposal/garbage collection, HDI of the municipality, age, education level, race/ethnicity.

Time varying variables: age of the mother, state of residence, number of antenatal visits, education level, year of conception, parity, receipt of conditional cash transfer benefit, marital status, and previous foetal loss

HDI: Human Development Index

IPW: Inverse Probability Weighting


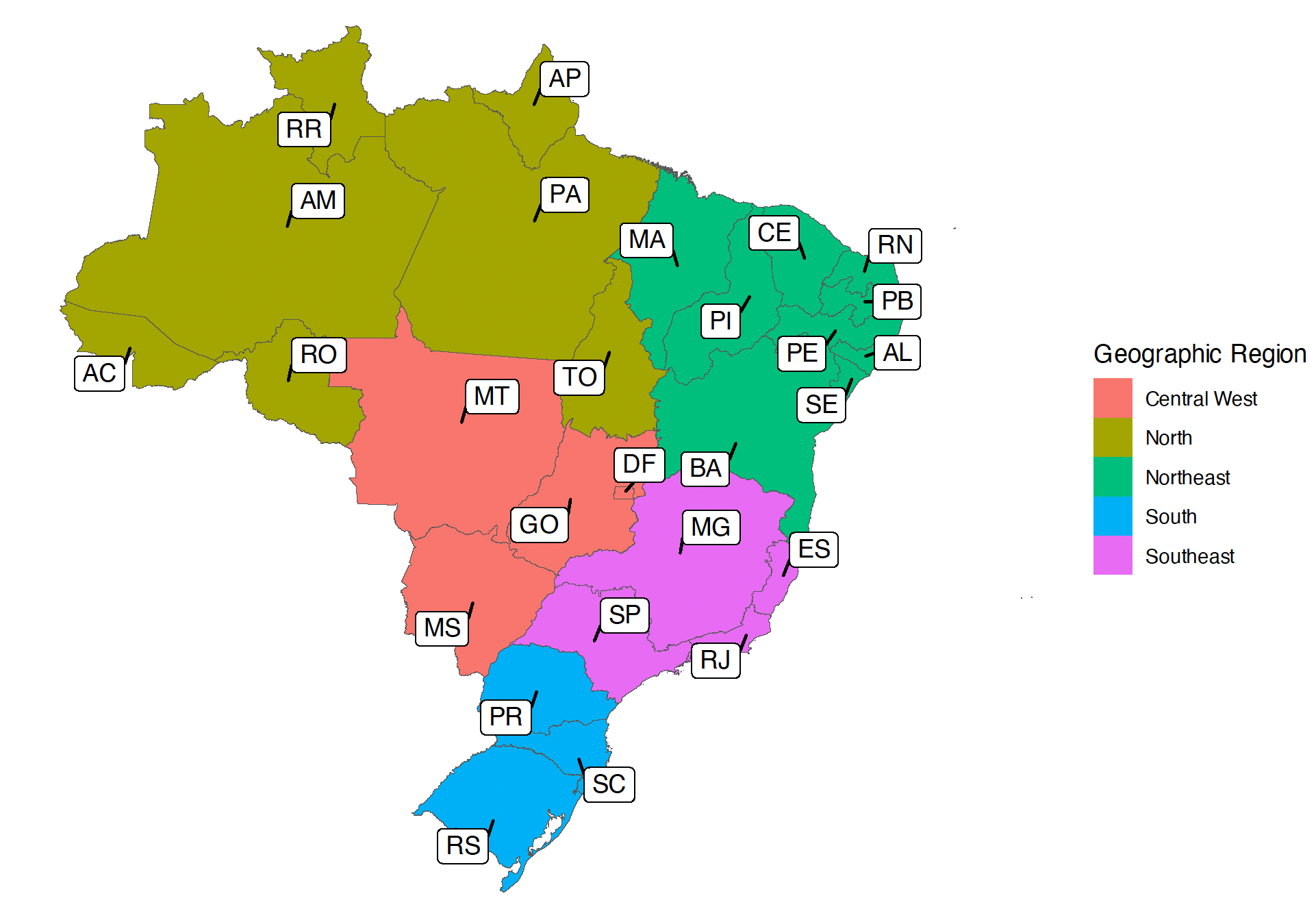


# **Supplementary Figure 1A**: Brazil’s map showing the geographic regions and States in each region.


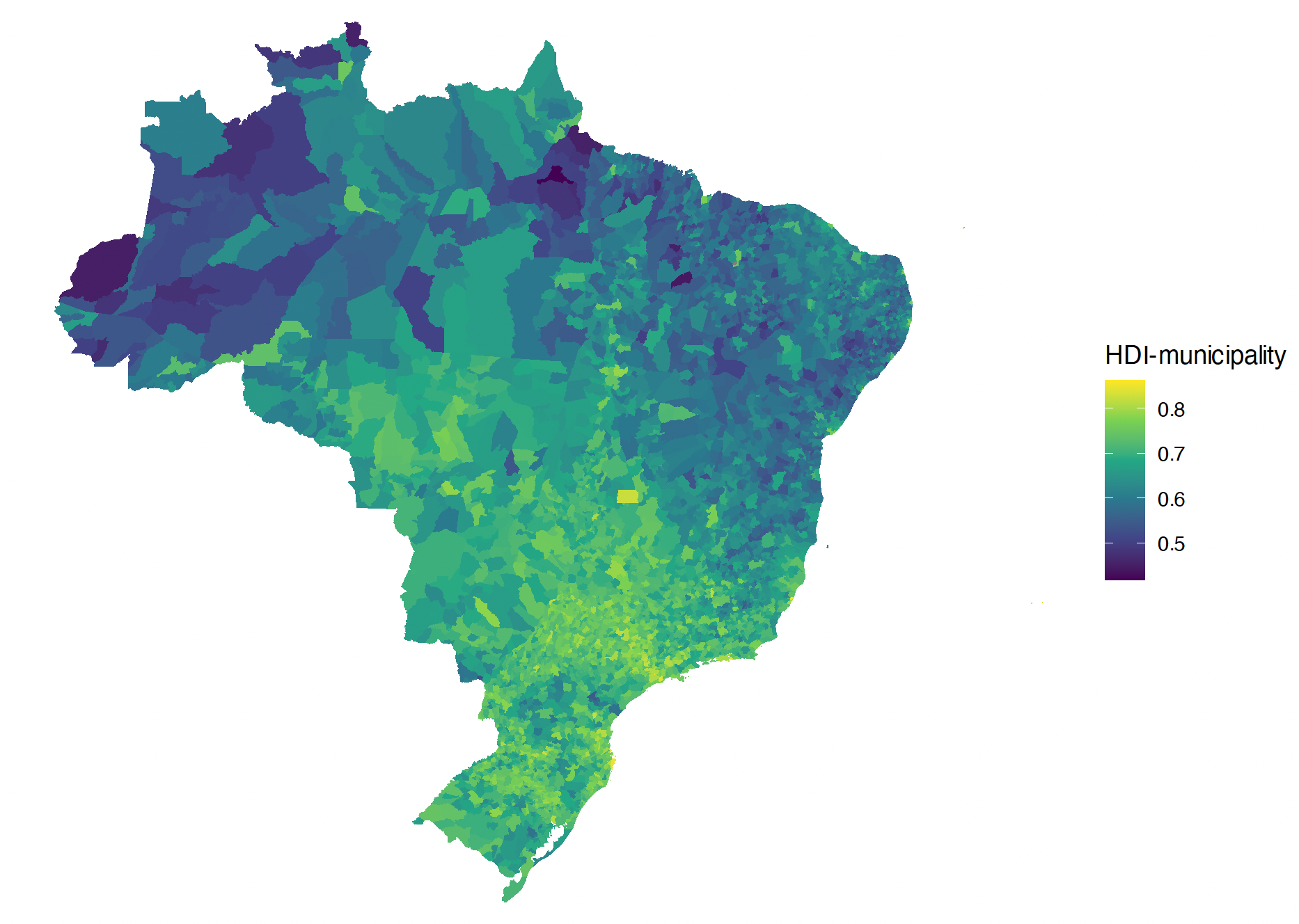


# **Supplementary Figure 1B**: Brazil’s map showing the human development index (HDI) of each municipality (5570)


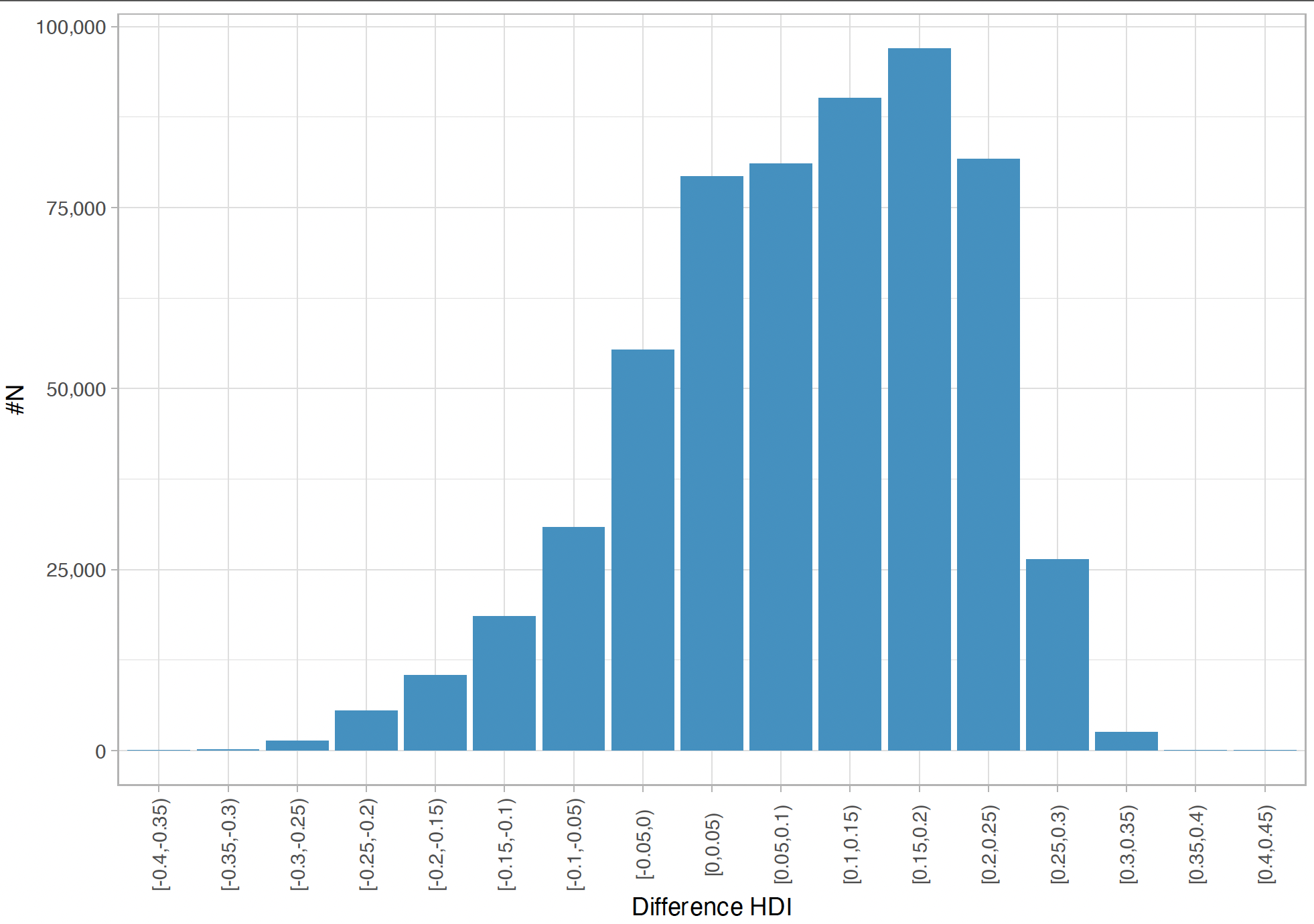


# **Supplementary Figure 2**: Histogram of the differences in human development index (HDI) of the municipalities of registration in the CadUnico and municipality of arrival (live birth system) in those classified as migrant.


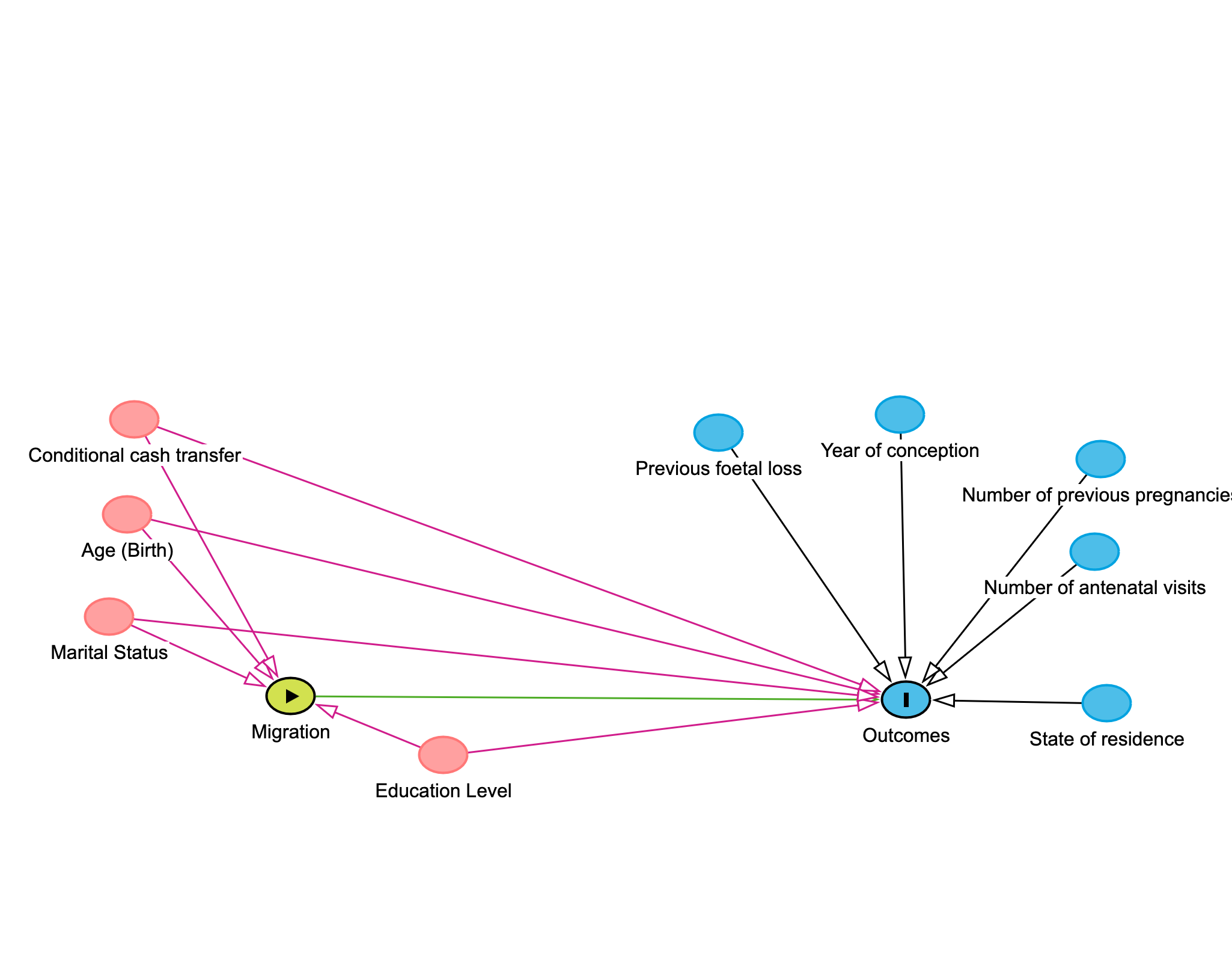


# **Supplementary Figure 3:** Directed acyclic graphic showing the assumed relationship between the variables in the outcome model. Outcomes evaluated: (i) timely initiation of antenatal care (at least one antenatal appointment within the first trimester), (ii) preterm (gestational age <37 weeks), (iii) low birth weight (LBW; birth weight <2500 g), (v) small for gestational age (SGA; weight below the 10th percentile for gestational age and sex), (vi) low Apgar score (Apgar score below 7 at 5 minutes after birth), (vii) congenital anomaly at birth, and (viii) neonatal mortality (death up to 28 days of life).


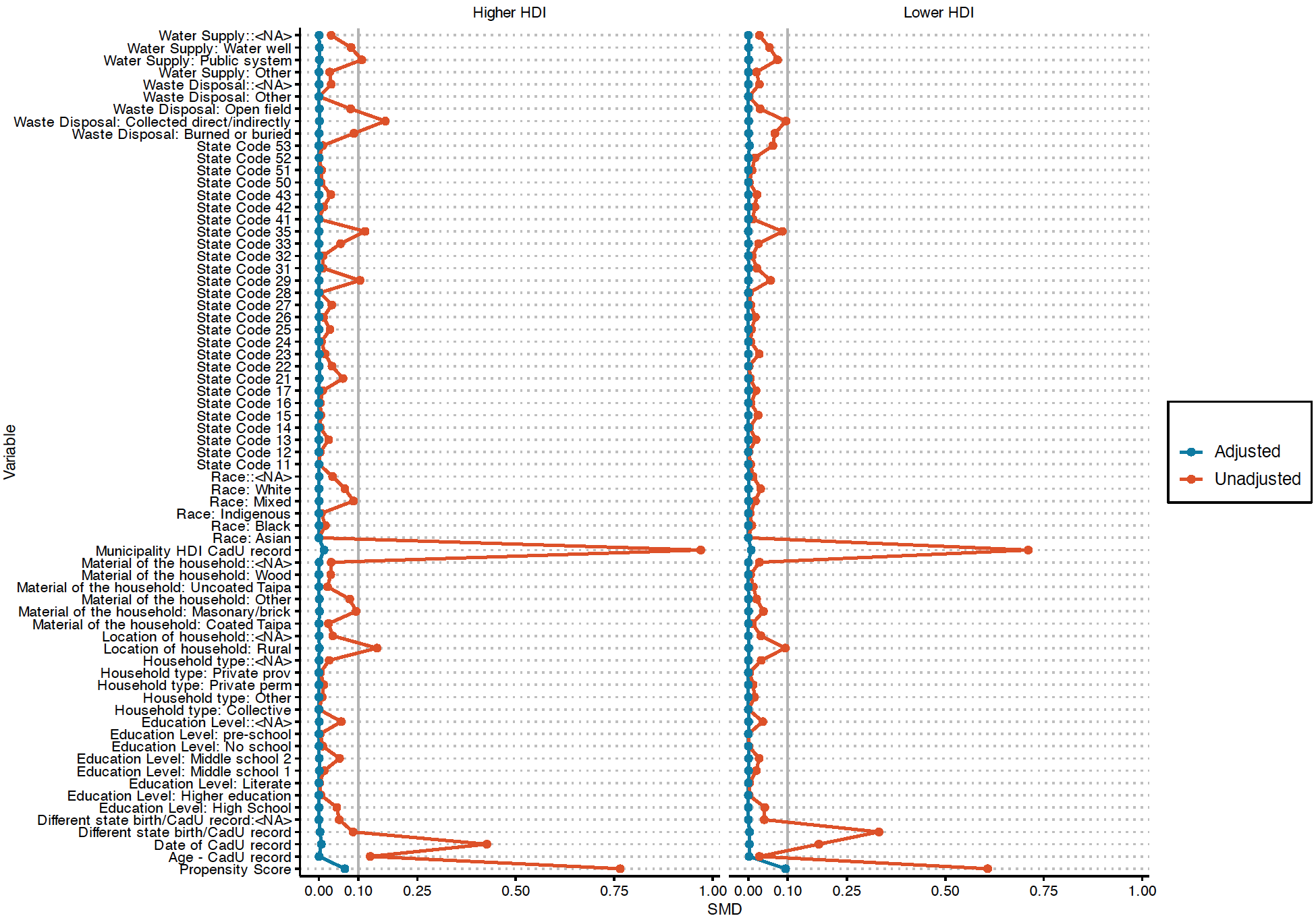


# Supplementary Figure 4**: Love plots representing the effect of the weighting in terms of standardized mean differences (SMD) among the weighted variables. CadU = CadUnico. Higher HDI = Equal/Higher HDI**


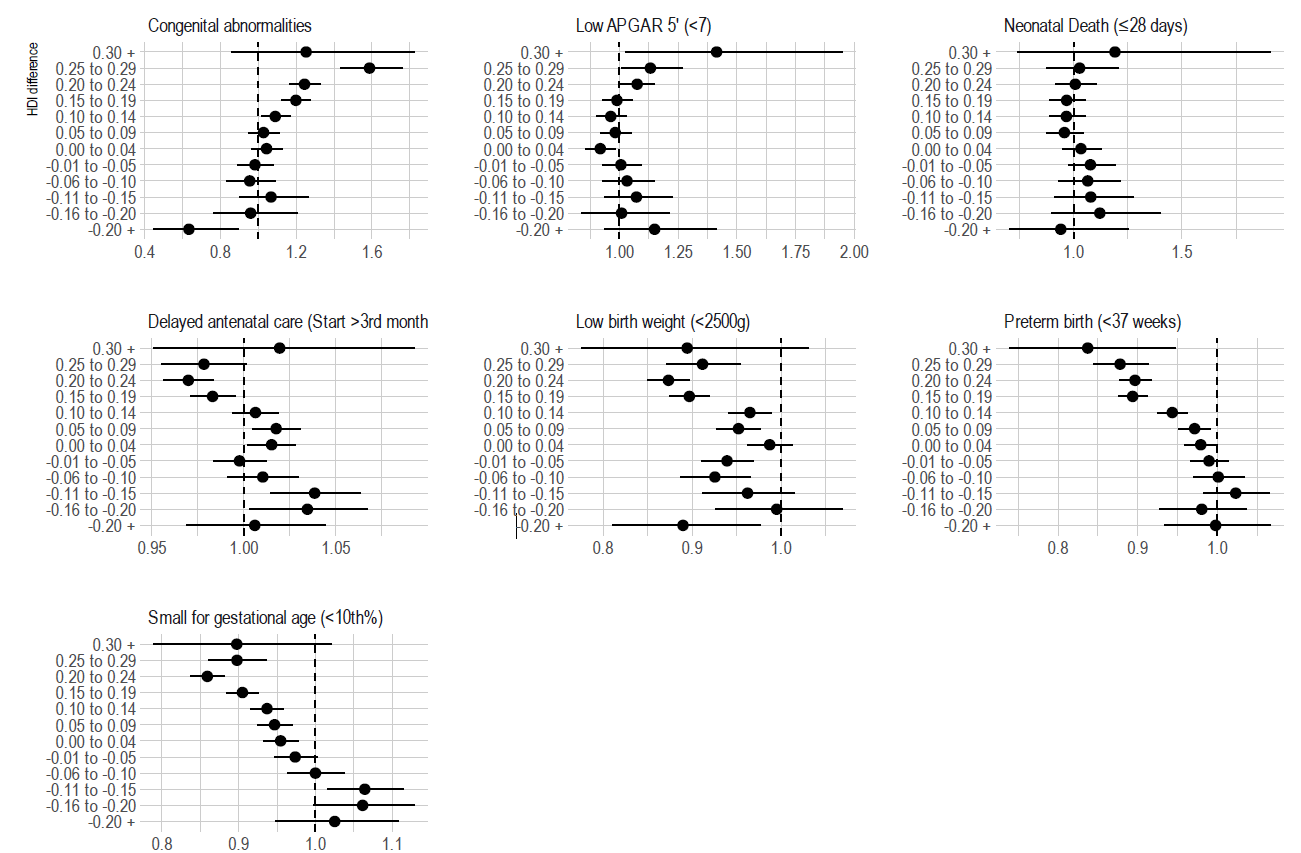


# Supplementary Figure 5: **Risk Ratio of perinatal outcomes of women according to migration by the difference in the Human Development Index (HDI) between municipality of origin and arrival. Positive values refer to migration to higher HDI, whilst negative values refer to migration to lower HDIs.**

# STROBE Statement—Checklist of items that should be included in reports of cohort studies

|  |  | **Recommendation** |  |
| --- | --- | --- | --- |
| **Title and abstract** | 1 | (*a*) Indicate the study’s design with a commonly used term in the title or the abstract | Present in the title |
|  |  | (*b*) Provide in the abstract an informative and balanced summary of what was done and what was found | Present in the abstract |
| **Introduction** | | |  |
| Background/rationale | 2 | Explain the scientific background and rationale for the investigation being reported | paragraph 3 - introduction |
| Objectives | 3 | State specific objectives, including any prespecified hypotheses | paragraph 4 - introduction |
| **Methods** | | |  |
| Study design | 4 | Present key elements of study design early in the paper | section study design and datasets - methods |
| Setting | 5 | Describe the setting, locations, and relevant dates, including periods of recruitment, exposure, follow-up, and data collection | section study design and datasets - methods |
| Participants | 6 | (*a*) Give the eligibility criteria, and the sources and methods of selection of participants. Describe methods of follow-up | section participants - methods |
|  |  | (*b*) For matched studies, give matching criteria and number of exposed and unexposed | N/A |
| Variables | 7 | Clearly define all outcomes, exposures, predictors, potential confounders, and effect modifiers. Give diagnostic criteria, if applicable | section outcomes - methods |
| Data sources/ measurement | 8* | For each variable of interest, give sources of data and details of methods of assessment (measurement). Describe comparability of assessment methods if there is more than one group | section study design and datasets - methods |
| Bias | 9 | Describe any efforts to address potential sources of bias | section Robustness checks: migration between pregnancies using a sibling analysis  and section Sensitivity analysis - methods |
| Study size | 10 | Explain how the study size was arrived at | N/A |
| Quantitative variables | 11 | Explain how quantitative variables were handled in the analyses. If applicable, describe which groupings were chosen and why | section statistical analysis - methods |
| Statistical methods | 12 | (*a*) Describe all statistical methods, including those used to control for confounding | section statistical analysis - methods |
|  |  | (*b*) Describe any methods used to examine subgroups and interactions | section statistical analysis - methods |
|  |  | (*c*) Explain how missing data were addressed | section statistical analysis - methods |
|  |  | (*d*) If applicable, explain how loss to follow-up was addressed | section statistical analysis - methods |
|  |  | (*e*) Describe any sensitivity analyses | section sensitivity analysis - methods |
| **Results** | | |  |
| Participants | 13* | (a) Report numbers of individuals at each stage of study—eg numbers potentially eligible, examined for eligibility, confirmed eligible, included in the study, completing follow-up, and analysed | first paragraph - results |
|  |  | (b) Give reasons for non-participation at each stage | figure 1 - results |
|  |  | (c) Consider use of a flow diagram | figure 1 - results |
| Descriptive data | 14* | (a) Give characteristics of study participants (eg demographic, clinical, social) and information on exposures and potential confounders | first paragraph - results |
|  |  | (b) Indicate number of participants with missing data for each variable of interest | table 1 - results |
|  |  | (c) Summarise follow-up time (eg, average and total amount) | NA |
| Outcome data | 15* | Report numbers of outcome events or summary measures over time | Table 1 - results |
| Main results | 16 | (*a*) Give unadjusted estimates and, if applicable, confounder-adjusted estimates and their precision (eg, 95% confidence interval). Make clear which confounders were adjusted for and why they were included | Paragraph 2 / figure 3/ Supplementary tables |
|  |  | (*b*) Report category boundaries when continuous variables were categorized | Paragraph 2 / figure 3/ Supplementary tables |
|  |  | (*c*) If relevant, consider translating estimates of relative risk into absolute risk for a meaningful time period | NA |
| Other analyses | 17 | Report other analyses done—eg analyses of subgroups and interactions, and sensitivity analyses | paragraph 4 and 5 - results |
| **Discussion** | | |  |
| Key results | 18 | Summarise key results with reference to study objectives | paragraph 1 - discussion |
| Limitations | 19 | Discuss limitations of the study, taking into account sources of potential bias or imprecision. Discuss both direction and magnitude of any potential bias | paragraph 7 - discussion |
| Interpretation | 20 | Give a cautious overall interpretation of results considering objectives, limitations, multiplicity of analyses, results from similar studies, and other relevant evidence | paragraph 3 - discussion |
| Generalisability | 21 | Discuss the generalisability (external validity) of the study results | paragraph 7 - discussion |
| **Other information** | | |  |
| Funding | 22 | Give the source of funding and the role of the funders for the present study and, if applicable, for the original study on which the present article is based | Role of funding source after methods - methods section |

*Give information separately for exposed and unexposed groups.

**Note:** An Explanation and Elaboration article discusses each checklist item and gives methodological background and published examples of transparent reporting. The STROBE checklist is best used in conjunction with this article (freely available on the Web sites of PLoS Medicine at http://www.plosmedicine.org/, Annals of Internal Medicine at http://www.annals.org/, and Epidemiology at http://www.epidem.com/). Information on the STROBE Initiative is available at http://www.strobe-statement.org.
